# Supplementary material for: Reconstituting the complete biosynthesis of D-lysergic acid in yeast
Source: Nat Commun. 2022 Feb 7;13:712. doi: 10.1038/s41467-022-28386-6 (PMC8821704; doi:10.1038/s41467-022-28386-6)
Supplement: Supplementary file 1 — Supplementary Information [file 41467_2022_28386_MOESM1_ESM.pdf]

SUPPLEMENTARY INFORMATION

Reconstituting the complete biosynthesis of *D*-lysergic acid in yeast

Garrett Wong[1,2,3,4]†, Li Rong Lim[1,2,3]†, Yong Quan Tan[1,2,3], Maybelle Kho Go[1,2,3], David J. Bell[4], Paul S. Freemont[4,5,6]*, and Wen Shan Yew[1,2,3]*.

[1] Synthetic Biology for Clinical and Technological Innovation, National University of Singapore, 28 Medical Drive, Singapore 117456, Singapore.

[2] Synthetic Biology Translational Research Programme, Yong Loo Lin School of Medicine, National University of Singapore, 14 Medical Drive, Singapore 117599, Singapore

[3] Department of Biochemistry, Yong Loo Lin School of Medicine, National University of Singapore, 8 Medical Drive, Singapore 117597, Singapore.

[4] Department of Infectious Diseases, Faculty of Medicine, Imperial College London, Exhibition Road, South Kensington, London SW7 2AZ, UK.

[5] London Biofoundry, Imperial College Translation & Innovation Hub, White City Campus, 80 Wood Lane, London W12 0BZ, UK

[6] UK Dementia Research Institute Care Research and Technology Centre, Imperial College London, Hammersmith Campus, Du Cane Road, London, W12 0NN

CONTENTS:

1. **Supplementary Discussion**

   Supplementary Discussion 1. Application of the EFI-EST for enzyme prospecting

   Supplementary Discussion 2. Adapting the tools for pathway-building in yeast

2. **Supplementary Methods**
3. **Supplementary Figures**
4. **Supplementary Tables**
5. **Supplementary References**

Supplementary Discussion

Supplementary Discussion 1. Application of the Enzyme Function Initiative – Enzyme Similarity Tool (EFI-EST) for enzyme prospecting.

The EFI-EST algorithm generates an analysis of protein similarity in the form of a protein sequence similarity network (SSN). Under the input option used in this study (Option A), the algorithm first performs a BLAST search of the input sequence against the Uniprot database for sequence hits at a user defined threshold (alignment E-value > 5, in this study). A second round of BLAST alignments are then performed on the returned hits, to obtain an "all-by-all" relational matrix. This relational matrix is visualized as the SSN, where each hit sequence is represented by a node in the network, and alignment scores greater than a user defined value are represented as the edges connecting the nodes[1].

This method provides a simplistic way of generating and visualizing the "all-by-all" multidimensional pairwise relationship. Where commonly used sequence similarity search methods (such as BLAST or other pairwise comparisons), would return a laundry list of pairwise alignments, this approach generates a relationship matrix that approximates the output from a multiple sequence alignment (MSA)[2]. The advantage of this method over MSA methods, lies in its ability to process larger datasets. MSAs and the phylogenetic analyses built on them, are computationally intensive endeavors; the required computational power for their analysis increases exponentially with the size of the dataset. The multidimensionality in the SSN can therefore be viewed as a computational heuristic to the analysis of phylogenetic relationships, trading a small degree of accuracy for higher processing speed. Furthermore, as the number of sequences analyzed increases, phylogenetic analyses tend to lose resolution as the alignment complexity increases[3]. SSNs on the other hand, get more reliable with increasing numbers of sequences, as the additional relationships help orientate correspondingly linked nodes within the network.

Interpretation of the SSN can thus be thought of as viewing a 3-dimensional phylogenetic tree from a perpendicular angle, in a top-down manner[4]. Viewing the SSNs at varying edge values can be thought of as analogous to viewing different focal planes down a phylogenetic tree, where different cluster topologies illustrate different sequence relationships[4]. Furthermore, the SSNs generated are able to tag additional pertinent information (such as phylogeny, PDB deposition, Swissprot annotation, Pfam, etc...) to each node that are searchable and sortable within Cytoscape[5]. The combined properties of the SSNs make them an ideal tool for various enzyme prospecting applications. By defining edges at suitable alignment scores, large collections of enzyme sequences can be fractionated into predicted isofunctional clusters. Cross-referencing nodes containing sequences that have been experimentally characterized, allows for a certain degree of functional assignment to other nodes within the same cluster[1,2,6].

In this work, we utilized these properties of the SSNs as a complement to other commonly used sequence similarity analyses, for two different outcomes: First, for the identification of correctly annotated protein sequences (EasE). Second, for the functional prediction of closely related isoforms (EasA and CloA). In both targeted outcomes, a known and characterized sequence was first used as the input for the EFI-EST algorithm. The generated SSN was then refined by adjusting the edge definition to an alignment score that corresponds to approximately 40% sequence identity. The alignment score cutoff that delineates the nodes into isofunctional clusters varies between protein families and must be determined by trial-and-error. This 40%-identity benchmark, serves as a useful starting point[1]. With this starting point, we adjusted the edge definition until

appropriately sized clusters could be discerned (Supplementary Fig. 1). The nodes within each cluster were then inspected for their associated annotations, such as but not limited to: Source organism, phylogenetic classification, PFAM and InterPro annotations, GO terms, EC, and availability in other databases such as PDB and SwissProt. From these associated annotations, sequences that have been experimentally validated or characterized can be identified and earmarked for cross-referencing clusters to putative functions.

For the first outcome, Nielson, et. al. (2014) hypothesized that the difficulty encountered trying to heterologously express EasE was likely due to mistakes made by the auto-annotation software when predicting amino acid sequences from genomic sequences[7]. Most of the protein sequences catalogued in the Uniprot database are derived from auto-annotations of genomic sequences. In eukaryotic genomes, the architecture of transcriptional units are more complex and may involve multiple introns and exons. In their report, Nielson, et. al., manually curated the predicted EasE sequences from *Aspergillus japonicus* (EasE_Aj) and *Aspergillus fumigatus* (EasE_Af) genomes and found that the Uniprot sequences were missing a short exon. They subsequently expressed the correctly predicted amino acid sequence that included the missing exon in *S. cerevisiae* and were now able to detect its activity.

In our approach, we postulated that the first neighbours of the EasE_Aj node, from its isofunctional cluster, would likely have the closest resemblance to the correctly predicted sequence. While a direct BLAST search of the sequence would also yield a list of its closest hits, the SSN allows us to approximate a MSA of the best hits from a larger pool of sequences to better identify sequences with the missing sequence segment included (Supplementary Fig. 1a). Eight such sequences of EasE orthologues were thus randomly selected from these criteria for screening. We further performed a MSA on the eight selected sequences with EasE_Aj and found that there was quite a substantial sequence divergence within the first half of the predicted FAD-binding domain (position 177-236 of EasE_Aj), as well as a four amino acid gap within the same region (Supplementary Fig. 2a). Of the eight screened sequences, EasE_Ec showed the most similarity to EasE_Aj across this region despite presenting the same gap as the other seven (Supplementary Fig. 2b). Curiously, EasE_Ec was also the only screened sequence to show detectable activity, albeit 10 times lower compared to EasE_Aj (Figure 2). We therefore hypothesize that the same exon was likely missed in the auto-annotation of the screened EasE orthologues. This hypothesis, however, was not pursued further as it was not the focus of our work and we had achieved our objective of identifying functional EasE orthologues for the reconstitution of the ergot alkaloid pathway.

For the second outcome, we sought to identify additional orthologues of EasA and CloA that catalyzed the required reactions for the eventual production of DLA in yeast. Both enzymes are known control points along the ergot alkaloid pathway and their isoforms are highly similar, with few key residues responsible for controlling their respective reaction outcomes. While at least one orthologue of EasA and CloA has been functionally expressed in heterologous hosts, these were usually performed in species closely related to their source organism. The objective was therefore in part to obtain redundancies in our pool of applicable parts for pathway construction in yeast.

EasA for instance, was shown by Cheng, et. al. (2010) to either be a reductase or isomerase variant, depending in part, on the presence of a F or Y residue at the 176 position[8]. In their work, they successfully switched the isomerase activity of EasA to one with reductase activity with a single F176Y point mutation. The reverse direction, however, was not as straightforward. The group proposed that while the F/Y residue at the 176 position is a crucial point for reductase activity, there were other unidentified components essential for isomerase activity. Our approach here, was

to attempt to account for these unidentified components with the SSN. We therefore used the EasA sequence from *Claviceps pupurea* (EasA_Cp), which was successfully expressed heterologously in *E. coli* by Cheng, et. al., to generate the SSN. From the EasA isofunctional cluster, we then randomly selected three additional sequences (from *Epichloe coenophialia*, *Neotyphodium lolii*, and *Periglandular ipomoeae*) on top of the sequence from *C. purpurea* (Supplementary Fig. 1b). As an added measure to ensure they were isomerase variants, we also performed a MSA of these four EasA orthologues together with the sequence from *Aspergillus fumigatus*, an established reductase variant. From this alignment, we verified the presence of the phenylalanine reside at the corresponding 176 position in our four selected sequences, and a tyrosine residue in the *A. fumigatus* control (Supplementary Fig. 3). These selected sequences when functionally screened, showed activity solely towards the agroclavine branch of the pathway (Figure 3).

Unlike the earlier discussed examples, CloA has never been heterologously expressed in a host organism from a different Order. There was thus no precedent sequence to use as an indication for accurate amino acid sequence prediction. The work around in this case, was to then select and screen more orthologues from each selected cluster. The use of the SSN in this case was therefore geared more towards the objective of trying to organize the sequence space of the CloA enzymes to make predictions on their product profiles.

Our goal in this endeavor was to identify CloA orthologs that would direct the flux of agroclavine to DLA. We therefore used the sequence identified from *Claviceps purpurea*, a well-established producer of DLA, to generate the CloA SSN. Unlike EasE and EasA, CloA belongs to the cytochrome P450 (CYP450) family of enzymes. The retrieved sequences predominantly had a sequence length withing the range of 490-530 amino acids and share a high degree of similarity owing to the heme binding domain (comprising around half the sequence length) (data not shown). We therefore had to limit the retrieved number of sequences to 5000 instead of 9000, in order to keep the generated SSN to a size that could be manipulated easily by a regular personal computer (up to 16 GB RAM). From the full network, we identified the cluster containing CloA sequences from organisms that have been shown to produce the ergot alkaloids (Supplementary Fig. 1c). This cluster was then further fragmented into five sub-clusters by increasing the alignment score. Amongst these five sub-clusters, one was found to be predominantly populated by sequences annotated as trichodiene oxygenases and was excluded from further analysis. Of the remaining four sub-clusters, we then selected 15 sequences that represented a random distribution between the sub-clusters for synthesis and screening (Supplementary Table 3). Of the 15 screened sequences, five orthologues were found to be able to produce varying quantities of DLA from supplemented agroclavine (Figure 4).

Through this exercise in searching for orthologous sequences, we have demonstrated the utility of the EFI-EST suite as a complementary tool to the pre-existing methods for *in silico* enzyme prospecting. Our approach provides a user-friendly and computationally simplistic method for navigating a larger sequence-function space before bringing on the existing well established bioinformatics tools to curate the derived results.

Supplementary Discussion 2. Adapting the tools for pathway-building in yeast

In order to quickly and easily assemble, and subsequently optimize, the ergot alkaloid pathway in yeast, we adopted the YeastFab Golden Gate system[9] for pathway construction in this work. Some modifications to the system were made to tailor it to better suit the requirements of rapid pathway reconstruction namely: (1) To enable assembly and screening of level 2 constructs in *E. coli* for higher throughput; (2) To allow for the integration of these constructs into any S288C-derived yeast strain.

Our modifications include a suite of pathway acceptor plasmids that allow both low and high-copy origins of replication (CEN and 2μ respectively) and auxotrophic markers (HIS3, LEU2, URA3), and an accompanying set of genome integration vectors for the retrotransposon sites: *YMRWδ15*, *YPRCδ15*, *YORWδ17* and two regions near autonomously replicating sequences (ARS): *ARS208a* and *ARS308a* (Supplementary Fig. 4).

We also established and characterized a simple toolbox of yeast genetic parts. This toolbox comprises of 30 promoters, most of which have been well-studied[10-13], and 21 terminators that were previously characterized[14]. The promoters were characterized first by the fluorescence output from the expression of yeCitrine (Supplementary Fig. 5), followed by the turnover of tryptophan into DMAT by the expression of DmaW (Supplementary Fig. 6). The extensions to the YeastFab system were tested by the introduction of DmaW and EasF into yeast, both as episomal constructs and as constructs stably integrated into the genome. The modified strains were assessed by their corresponding capability to produce DMAT and 4DMA (Supplementary Fig. 7 and 8). DMAT and 4DMA are not commercially available. We therefore validated the detection of these compounds by their corresponding *in vitro* reactions using enzymes purified from *E. coli* expression.

Having demonstrated the successful use of the expanded YeastFab system in a simple 1-2 transcriptional unit system, we subsequently applied it to the construction of our engineered strain (Supplementary Fig. 14).

SUPPLEMENTARY METHODS

Assembly of modified YeastFab plasmids

The pathway acceptor plasmids and genome integration plasmids were constructed through Gibson Assembly[15]. Pathway acceptor plasmids were assembled from four fragments, each fragment holding some core elements (Kan^R and ColE1 origin, RFP expression cassette with insert and release sites, a yeast origin of replication, and a yeast selection marker). Genome integration plasmids were assembled similarly from five fragments (URA3 selection marker flanked by URR sites, RFP expression cassette with insert sites, Amp^R and ColE1 origin, upstream and downstream genome integration homology regions). All fragments were created through PCR amplification (Takara PrimeSTAR™) from appropriate sources. The created plasmids were verified by restriction digest and Sanger sequencing.

Bioinformatics analysis

All SSNs used in this study were generated using a protein sequence query for the initial BLAST search (Option A), with the parameters set to retrieve a maximum of 9000 sequences (except for CloA, where the maximum was set at 5000 sequences) with a minimum alignment E-value of 5, through the EFI-EST webtool[2]. The initial network was calculated by defining an edge to represent a relationship with an alignment score greater than or equal to the equivalent of 40 % sequence identity. Each SSN was subsequently individually refined by increasing the edge score until the clusters fragmented into smaller hypothetical iso-functional clusters. Manipulation and visualization of SSNs were performed using the Cytoscape software[5]. Selected sequences were then retrieved from the Uniprot database using the associated Uniprot numbers from the SSN

Multiple sequence alignments were performed using the Clustal Omega software, accessed through the Uniprot website[16].

^{13}C-Labelled experiments with ^{13}C-2-indole-*L*-tryptophan

A stock solution of ^{13}C-2-indole-*L*-tryptophan (Sigma) was prepared by dissolving the powder in 20% (w/v) galactose to a final concentration of 10 mM and filter-sterilized. ^{13}C-Labelling of DLA and its associated pathway intermediates were carried out using the same protocol described for the production of ergot alkaloids but were induced with the stock solution of ^{13}C-2-indole-*L*-tryptophan in galactose instead of just galactose alone, to a final concentration of 1 mM ^{13}C-2-indole-*L*-tryptophan and 2% (w/v) galactose. The negative control used was a similar preparation with *L*-tryptophan (Sigma) in place of ^{13}C-2-indole-*L*-tryptophan.

Flow cytometry analysis of yeast promoter library

The promoter reporter plasmid pGlo3 containing the promoter library inserts were transformed into yeast BY4741 cells. From the transformants, three individual colonies were picked and cultured in liquid SC-URA for 30 h at 25 °C, 220 rpm until saturation (OD ≈ 3). Subsequently, fresh media was inoculated with saturated cell culture in 1:200 dilution and grown for 12 h at 25 °C, at which point the optical density at 600 nm (OD_{600}) reached approximately 0.9 to 1.2, which corresponds to the exponential growth phase. To 180 µL of ice-cold PBS was added 20 µL of the

cell culture and the 96-well plate holding the samples was kept at 4 °C until flow cytometry analysis, which was no longer than 4 h later. The remaining cell cultures were grown for a further 6 h at 25 °C until the OD_{600} reached ~ 2.0 – 2.5, corresponding to early stationary phase. Similarly, 20 μL of the culture was diluted in 180 μL ice-cold PBS and kept cold until analysis.

The yeGFP and mKOκ emissions of each individual cell were measured using the BD Accuri™ flow cytometer. For each sample, 20 000 cells were measured, and the flow rate was adjusted to roughly 2000 cells·s^{-1}. For each batch of samples, a strain expressing P_{PGK1} driven yeGFP and another strain expressing mKOκ on high copy *2μ* plasmids were included as fluorescence compensation controls and a strain containing plasmid pCKU (which does not express any fluorescent protein) served to account for background fluorescence. Results were analyzed by using the FlowJo (Version 10) software. Fluorescence bleed-through between the green and orange emission channels were first compensated for using the *2μ* plasmid controls. Subsequently, the signal readout from promoter activity was obtained as the geometric means of the orange emissions of the plasmid harboring strains (identified by yeGFP emission) minus the background orange emission as measured by using the pCKU strain.

Expression and purification of DmaW and EasF from *E. coli* for in vitro assays

The genes encoding these two proteins were cloned into pET15B vectors and recombinantly expressed in *E. coli* BL21(DE3) cells. Expression was carried out by growing cells in 2YT media to an OD_{600} of 0.7 at 37 °C, prior to induction with IPTG (2 mM) at 20 °C for 20 hours. The cells were then pelleted by centrifugation at 4 °C, 2236 x g for 10 mins. Pelleted cells were resuspended in binding buffer (5 mM imidazole, 0.5 M NaCl, 20 mM Tris-HCl, pH 7.9) at 4 °C and lysed *via* sonication. Cell debris was then clarified by centrifugation at 10956 x g for 20 mins at 4 °C. All subsequent purification steps were done at 4 °C. The clarified cell supernatant was then added to 200 *μ*L of Ni^{2+}-NTA chelating sepharose resin equilibrated in binding buffer. This was incubated for 30 mins with shaking at 140 rpm. The resin was washed three times with 2 mL wash buffer (60 mM imidazole, 0.5 M NaCl, 20 mM Tris-HCl, pH 7.9) for 5 mins with shaking at 90 rpm for each washing step. The bound protein was eluted twice from the resin with 500 *μ*L of His-Elute buffer (100mM L-histidine, 0.5M NaCl, 20 mM Tris-HCl, pH 7.9) for 10 mins with shaking at 70 rpm. The fractions were analyzed using SDS-PAGE and those containing the protein(s) were concentrated using 3 kDa molecular weight cut-off (MWCO) Ultra-0.5 spin filters (Amicon). The purified solution was then dialyzed against the storage buffer containing 50 mM Tris-HCl, pH 7.5, 5 mM $CaCl_2$, 50% glycerol and stored at -20 °C.

The *in vitro* biosynthesis of DMAT was prepared in a 60 *μ*L reaction volume containing 50 mM Tris-HCl, pH 7.5, 5 mM $CaCl_2$, 1 mM *L*-tryptophan, 1 mM DMAPP, and 10 *μ*L purified DmaW. The reaction was incubated at 30 °C for 18 hrs. The reaction was stopped by filtering off the enzyme using a 3 kDa MWCO Ultra-0.5 spin filters (Amicon). The sample was either stored at -20 °C or immediately analysed using LC-MS. The *in vitro* biosynthesis of 4DMA was prepared similarly but with the addition of 1 mM SAM and 10 *μ*L purified EasF.

Nuclear Magnetic Resonance (NMR) analysis of biosynthesized DLA

Cell culture to produce DLA for NMR analysis was performed as described for the small scale production of ergot alkaloids in the main text. DLA was purified from the culture media (8 L) by

first lyophilizing the collected clarified media. The dried culture was subsequently reconstituted in a 300 mL of ddH_2O and purified by liquid chromatography using an AKTA Pure 25M (Cytiva) affixed with a C-18 preparative column (Agilent Zorbax Eclipse XDB-C18, semi-preparative; 9.4 X 250 mm, 5 μm particle size). The mobile phases used consisted of: A, water with 0.1% trifluoroacetic acid; and B, acetonitrile with 0.1% trifluoroacetic acid. Semi-preparative chromatography was carried out over a constant flow rate of 2 mL/ minute, 2 mL injections, with a stepped gradient as follows: 90%A/ 10% B for 10 minutes, 90% A/ 10% B to 80% A/ 20% B over 50 minutes. Between runs, the column was washed with 100% B for 4 column volumes (CV) (80 mL) of 100% B at a flow rate of 10 mL/min, before re-equilibrating to 90% A/ 10% B for 4 CV at a flow rate of 2 mL/min. Elution of DLA was monitored by absorbance at 310 nm, fractions corresponding to peaks at 310 nm were collected and pooled. The pooled fractions were concentrated by lyophilization and 150 μL aliquots were taken for LC-MS/MS analysis of the purity and confirmation of the presence of DLA. The remaining pooled fractions were lyophilized to dryness and stored at -20 °C.

The sample for NMR analysis was prepared by adding 2 mL of D_2O (Sigma) to the combined dried fractions, any insoluble material was removed by centrifugation at 2236 x g, 20 minutes. Subsequently, 1 mL of D_2O saturated with the sample was used for analysis of the 1H-NMR spectra using a Bruker AVANCE 500 MHz NMR spectrometer at the Department of Chemistry, National University of Singapore.

SUPPLEMENTARY FIGURES

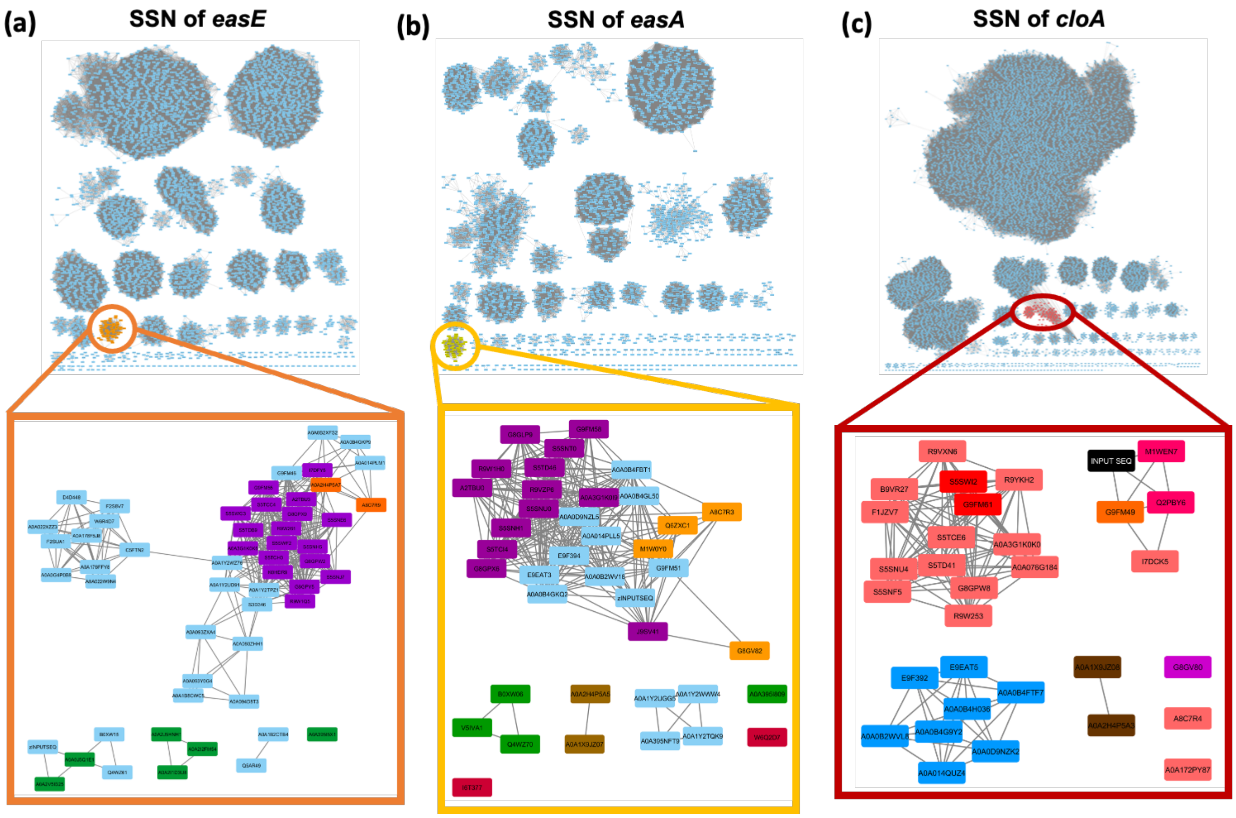

Supplementary Fig. 1. Sequence Similarity Networks (SSN) generated from known gene targets of the ergot pathway using the EFI-EST webtool[1,6]. In the representation of an SSN, each sequence surveyed is denoted as a node and is linked to other relevant nodes by edges. Each edge represents a predefined degree of similarity. By defining an appropriate alignment score as an edge, nodes can be delineated into isofunctional clusters that group related sequences together in a similar way a multiple sequence alignment draws a consensus sequence. Enzyme sequences within isofunctional clusters could then be expected to be capable of catalyzing similar reactions. Uncharacterized enzymes that are closely related to a known target, could then be identified, and tested for desired qualities. Expanding on the hypothetical isofunctional clusters of EasE, EasA, and CloA allows for the further delineation of the more closely related sequences for the better prediction of their specific activity. (a) Expanded SSN of the EasE isofunctional cluster. Nodes are colour coded by the genus from which the sequences come from; purple: *Epichloe*, orange: *Claviceps*, green: *Aspergillus*, and blue: mainly *Penicillium*, *Pseudogymnoascus*, and *Trichophyton*. (b) Expanded SSN of the EasA isofunctional cluster. Reductase variants of EasA showing more sequence divergence and fractioning away from the main cluster. Node colour denote genus of the source organisms; purple: *Epichloe*, orange: *Claviceps*, green: *Aspergillus*, red: *Penicillium*, brown: *Claviceps gigantea* and *africana*, blue: others (c) Expanded SSN of the cluster containing the known ergoline-C17 oxidases. Sub-clusters are grouped by the known product profiles of closely related source organisms.

(a) **Uniprot ID**

```
S5TDB9      S5TDB9_EPINE       170  PDSLQIHTYFLKDIHYDDNFLVHGDAT----GSGPAVTLGAGVVHSEVYKHGIDHKYSVV  225
R9W1Q5      R9W1Q5_9HYPO       170  PDSLQIHTYFLKDIHYHDNFFAQGDAT----GSGPAVTLGAGVMHSEVYKHGIDHKYSVV  225
G9FM46      G9FM46_9HYPO       168  PNSFQIHTNLLKSISFHENFVARGSTT----CSGPAVTLGAGVMHWEVYAHGVENGYTIV  223
K0HDR9      K0HDR9_EPIEL       170  PDSLQIHTYFLKDIHYHDNFFAQGDAT----GSGPAVTLGAGVMHSEVYKHGIDHKYSVV  225
I7DFY5      I7DFY5_9HYPO       168  PHSFQIHTNLLKNISFHENFVAGGSTT----GYGPAVTLGAGVMHWEVYAHGAEKGYTIL  223
A8C7R9      EASE_CLAFS         169  PHSFQISTSSLKTISLHENFVPRGSTT----GHGPAVTLGAGVMQWEVYAHGVKNAYTIL  224
A0A0S7DKV5  A0A0S7DKV5_9EURO   165  PDSFQIHTHRMKQIEYHDNFRAVSSDI----DQGPAVSVGAGVLLGEMYARGARDGWVVV  220
R9W261      R9W261_EPICN       170  PDSLQIHTYFLKDIHYHDNFLVHGDAT----GSGPAVTLGAGVVHSEVYKHGIDHKYSVV  225
easE_Aj                        177  PDSVEIHTHHLNSVQYHPNFRPAGSSERQSAPGQPAVTVGAGILLGDLYARGASEGWIVV  236
                                    *.*.:* *  :: :  . **   ..         ***::***::  ::* :*  . : ::
```

(b) **Uniprot ID**

```
R9W261      R9W261_EPICN       170  PDSLQIHTYFLKDIHYHDNFLVHGDAT----GSGPAVTLGAGVVHSEVYKHGIDHKYSVV  225
easE_Aj                        177  PDSVEIHTHHLNSVQYHPNFRPAGSSERQSAPGQPAVTVGAGILLGDLYARGASEGWIVV  236
                                    ***::***:.*:.::** **   *.:      . ****:***:: .::* :* .. : **
```

Supplementary Fig. 2. Multiple sequence alignment of the FAD-binding domain from (a) the eight selected EasE orthologues showing the most sequence divergence and a four amino acid gap. (b) Pairwise alignment of the same region in (a) for the two EasE orthologues (EasE_Ec and EasE_Aj) that showed activity in our screen. Uniprot ID legend: S5TDB9 – *Neotyphodium lolii*; R9W1Q5 – *Epichloe funkii*; G9FM46 – *Periglandular ipomoeae*; K0HDR9 – *Epichloe elymi*; I7DFY5 – *Epichloe inebrians*; A8C7R9 – *Claviceps fusiformis*; A0A0S7DKV5 – *Aspergillus lentulus*; R9W261 – *Epichloe coenophialia*; easE_Aj – *Aspergillus japonicus*.

Uniprot ID

```
S5TCI4    117   ANGFDLVSSSAVPVSPGEPTPRALSDDEINSYIGDFVQAAKNAVLEAGFDGVELHGANGF   176
G9FM51    117   SNGLELKSSSEVPVAPGEPTPRPLSEEEIQSYIFDYAQGAKNAVHEAGFDGVEIHGANGF   176
R9W1H0    117   ANGFDLVSSSAVPVSPGEPTPRALSDDEINSYIGDFVQAAKNAVLEAGFDGVELHGANGF   176
M1W0Y0    117   SQGLKLESSSEVPVAPGEPTPRALDEDEIQQYILDYVQGAKNAVHGAGFDGVEIHGANGF   176
Q4WZ70    121   D-MKDLISSSAVPVEEKGPLPRALTEDEIQQCIADFAQAARNAI-NAGFDGVEIHGANGY   178
                    .* *** ***    * ** * ::**:. * *:.*.*:**:  *******:*****:
```

Supplementary Fig. 3. Multiple sequence alignment the selected EasA sequences showing the 60 amino acid region containing the identified critical residue. Residues highlighted in red indicate the 176 position, showing the presence of a phenylalanine residue in the selected isomerase variants and a tyrosine residue in the reductase variant control. Uniprot ID legend: S5TCI4 – *Neotyphodium lolii*; G9FM51 – *Periglandular ipomoeae*; R9W1H0 – *Epichloe coenophialia*; M1W0Y0 – *Claviceps purpurea*; Q4WZ70 – *Aspergillus fumigatus*.

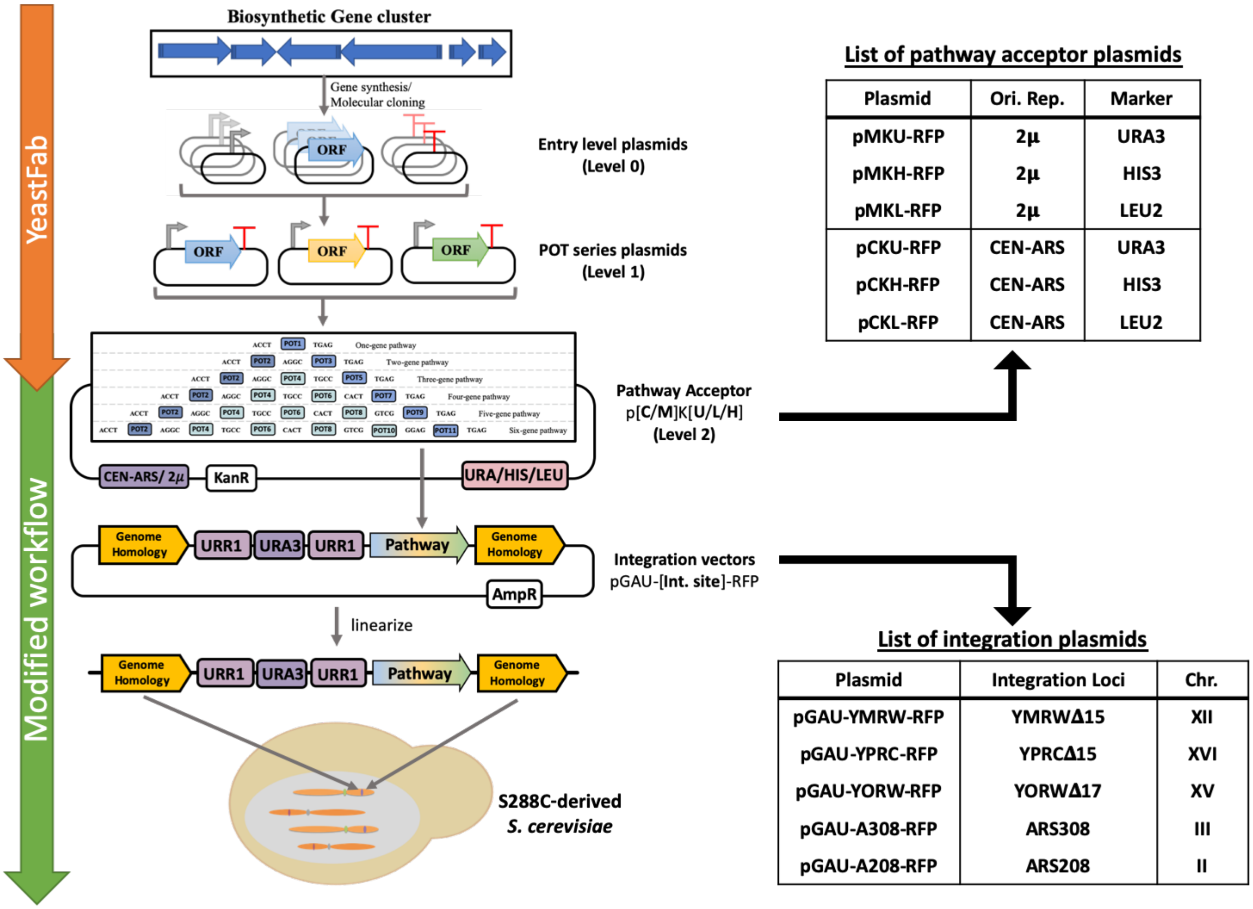

List of pathway acceptor plasmids

Plasmid	Ori. Rep.	Marker
pMKU-RFP	2μ	URA3
pMKH-RFP	2μ	HIS3
pMKL-RFP	2μ	LEU2
pCKU-RFP	CEN-ARS	URA3
pCKH-RFP	CEN-ARS	HIS3
pCKL-RFP	CEN-ARS	LEU2

List of integration plasmids

Plasmid	Integration Loci	Chr.
pGAU-YMRW-RFP	YMRWΔ15	XII
pGAU-YPRC-RFP	YPRCΔ15	XVI
pGAU-YORW-RFP	YORWΔ17	XV
pGAU-A308-RFP	ARS308	III
pGAU-A208-RFP	ARS208	II

Supplementary Fig. 4. Modifications made to the YeastFab assembly system for this project. Two sets of plasmids were created to improve on the overall workflow: the first was a series of pathway acceptor vectors (level 2) that enables for the screening of pathway modules created to be screened for in *E. coli* and quickly tested as episomal plasmids in yeast. The second was a series of yeast genome integration vectors to expand the repertoire of integration sites available. The URA3 marker on the integration fragments are designed to be flanked by the homologous sequence URR1, this allows for the removal of the marker by homologous recombination upon the counter-selection with 5-fluoroorotic acid.

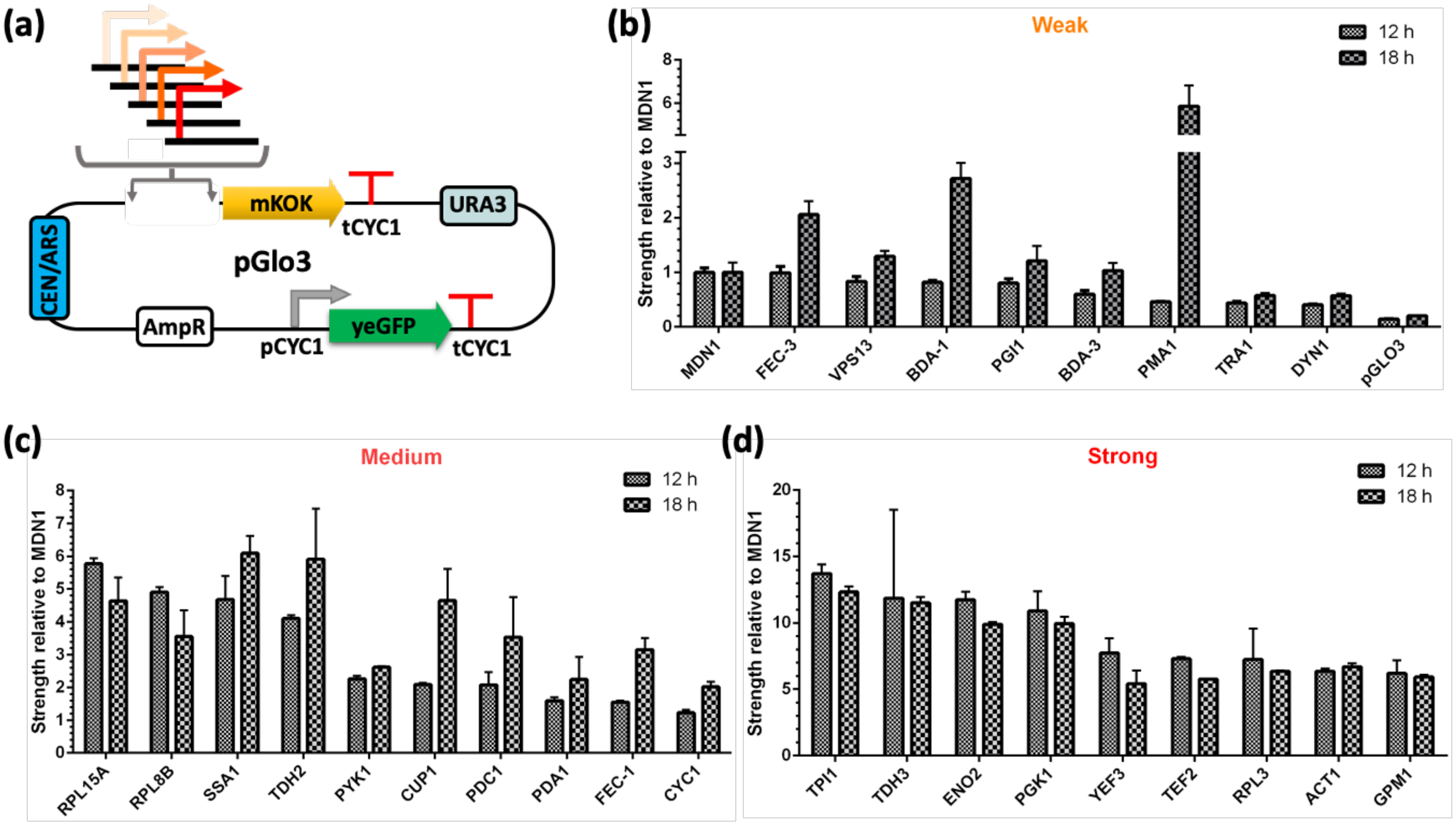

Supplementary Fig. 5. Promoter strength measurements at 12 and 18 hours of growth, arranged in descending order by their strengths at 12 hours. (a) Schematic representation of the promoter reporter plasmid, pGLO3. A promoter released from HCKan_P displaces the RFP cassette in a Golden Gate reaction with Esp3I. When transformed into yeast, the promoter drives the expression of mKOK (orange fluorescent protein). The P_{CYC1}-yeGFP-T_{CYC1} cassette serves to differentiate cells that harbour the plasmid from those that do not. (b) Weak promoters – relative strengths to P_{MDN1} of 1 and below, at 12 hours of growth. (c) Medium strength promoters – relative strengths between 1 to 6. (d) Strong promoters – defined by having ≥6 times the strength of P_{MDN1}.

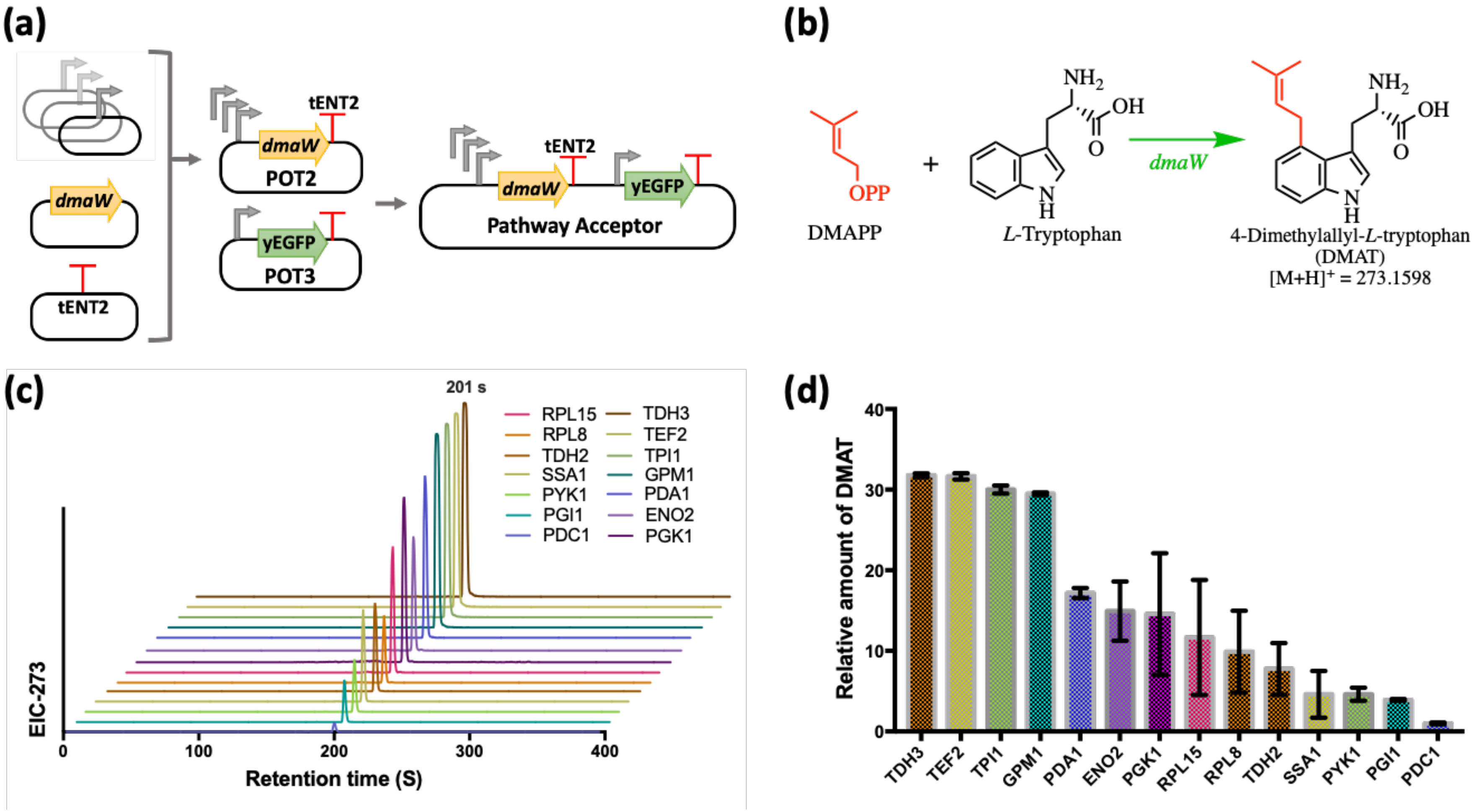

Supplementary Fig. 6. Testing the pathway acceptor plasmids and validating promoter strength data by the production of DMAT. (a) Illustration of the assembly process to generate pMKU-DmaW series of plasmids. (b) The first reaction of the ergot alkaloid pathway, catalysed by DmaW producing DMAT. (c) Overlaid LC-MS chromatograms for 273 m/z of the analysed samples showing peaks of varying sizes corresponding to the amount of DMAT produced. (d) Comparison of the relative amounts of DMAT produced, estimated from the peak area response of the extracted ion chromatograms. Error bars calculated from three biological replicates.

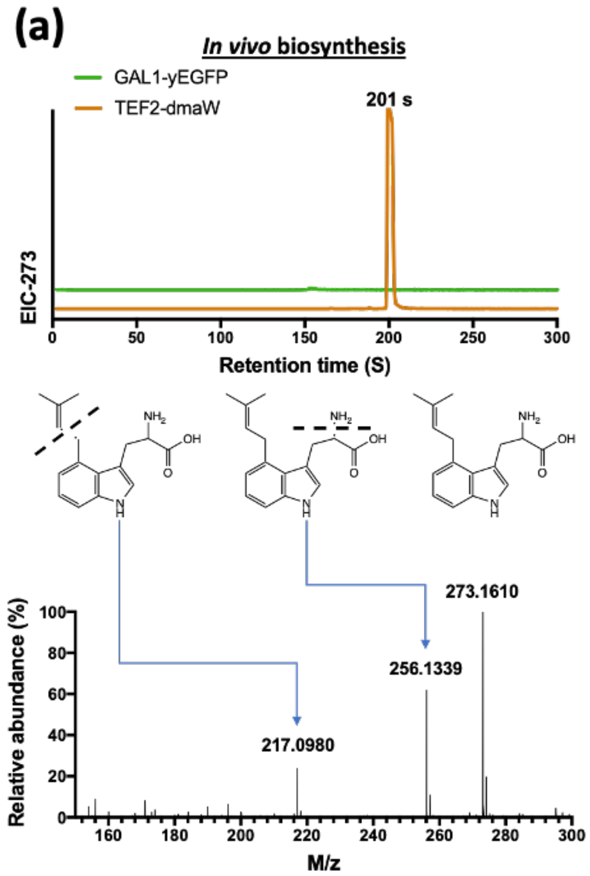

Supplementary Fig. 7. Validating the production of DMAT produced from the pMKU-DmaW series of plasmids. Comparison of the retention time and mass spectra of DMAT produced *in vivo* (a) and *in vitro* (b) from purified enzymes fed with DMAPP and tryptophan. Proposed structures of the simplest fragment ions observed.

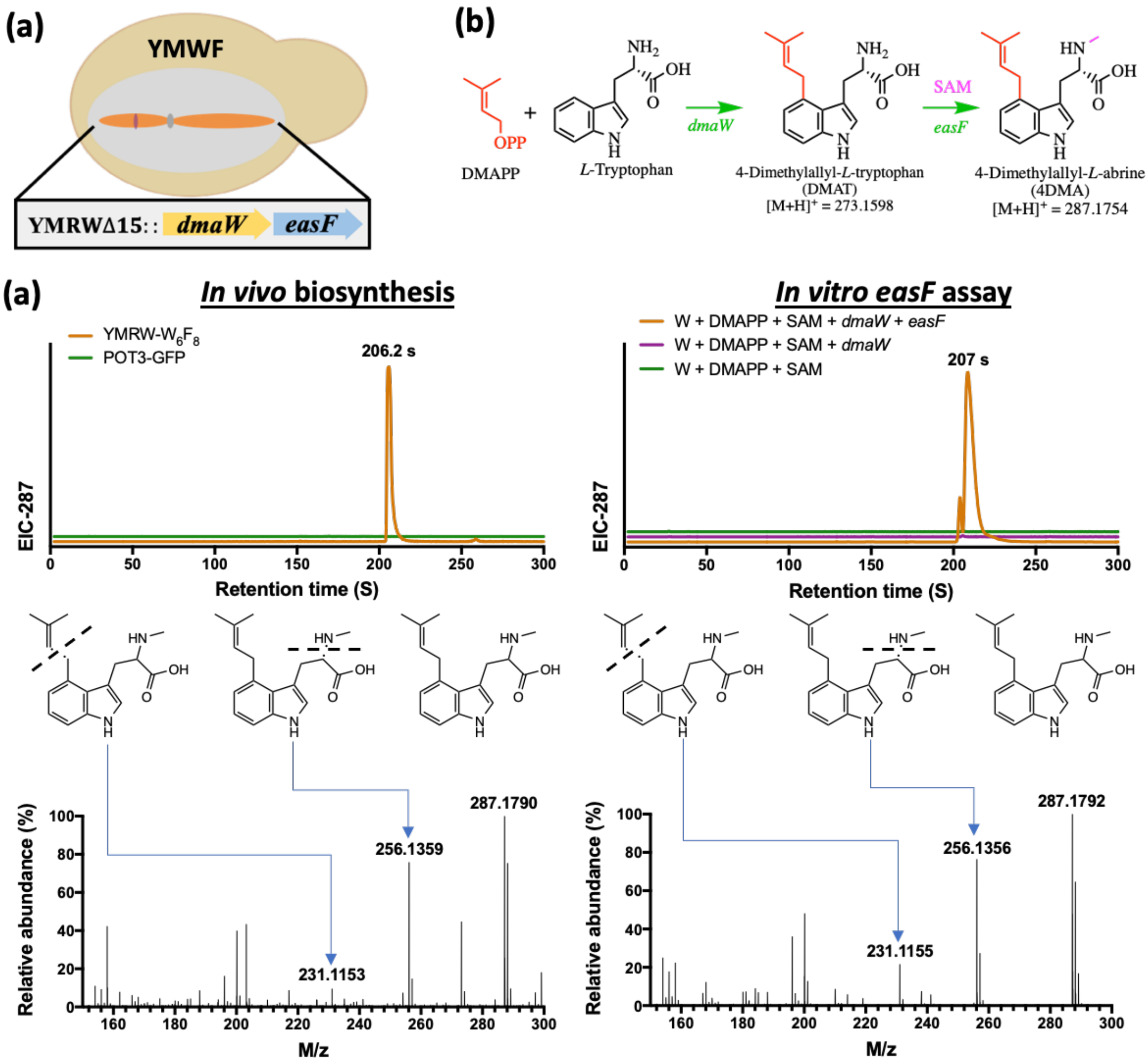

Supplementary Fig. 8. Testing the genome integration vectors via the production of 4DMA. (a) Cartoon representation of the strain (YMWF) created, containing the expression cassettes: P_{TEF2}-DmaW-T_{ENT2} and P_{GPM1}-EasF-T_{PRX1} integrated onto the *YMRWΔ15* transposon site. (b) Reactions catalysed by DmaW and EasF to produce 4DMA from tryptophan and DMAPP. Comparison of the retention time and mass spectra of 4DMA produced *in vivo* (c) and *in vitro* (d) from purified enzymes supplemented with DMAPP, tryptophan, and S-adenosyl methionine (SAM). Proposed structures of the simplest fragment ions observed.

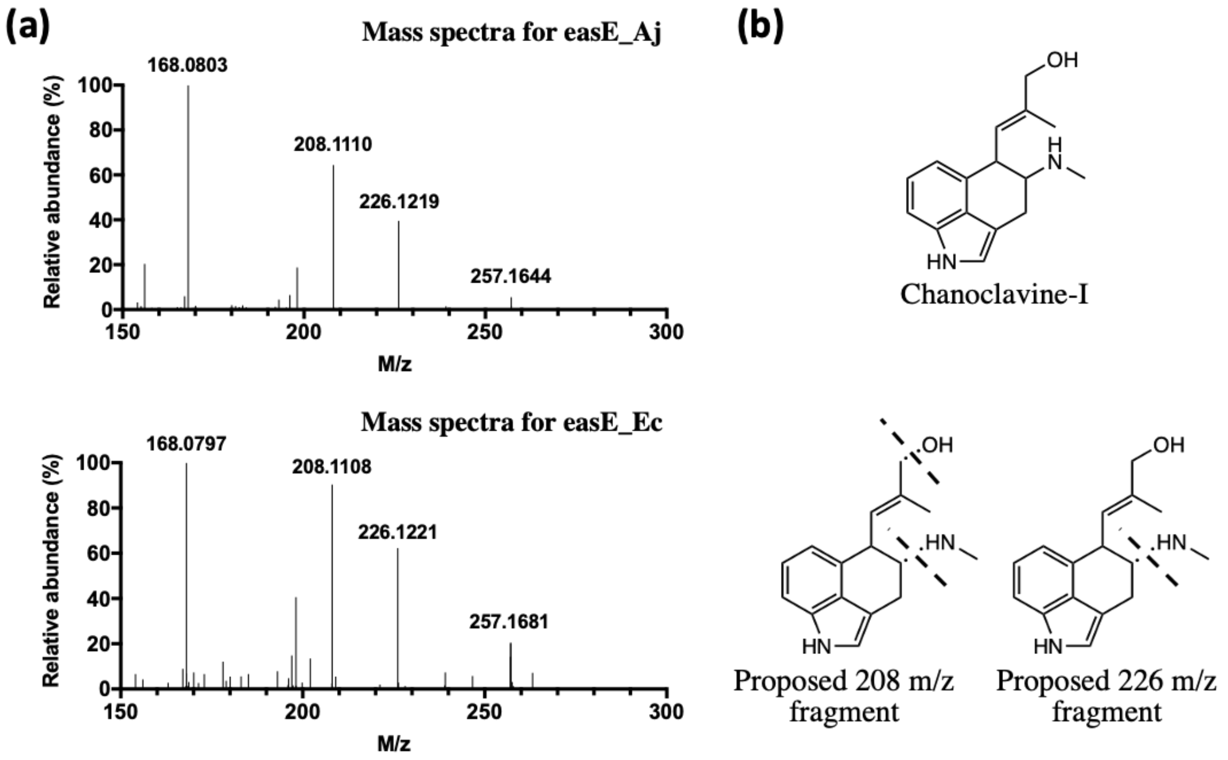

Supplementary Fig. 9. MS/MS fragmentation spectra of the peaks eluted at 120.6 seconds from the screen of EasE orthologues demonstrating the same product being eluted from both (a) EasE_Aj and EasE_Ec. (b) Proposed structures for the two simplest fragment ions, 226 and 208 m/z.

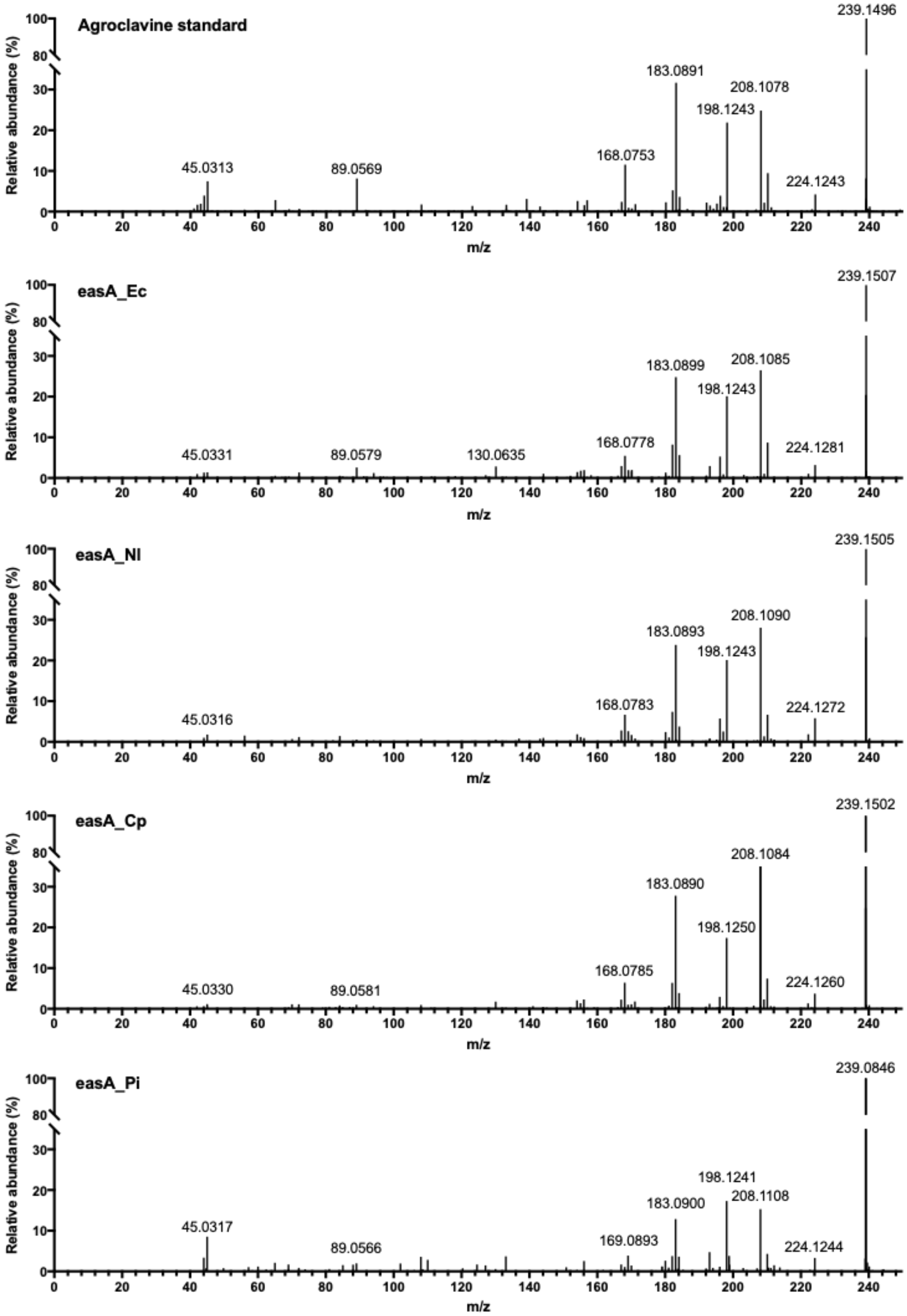

Supplementary Fig. 10. MS/MS fragmentation spectra of the peaks eluted at 149.6 seconds from the screen of EasA orthologues. Comparison of the fragmentation patterns against the commercial agroclavine standard demonstrates the production of agroclavine in strains with the EasA_Ec, EasA_Nl, and EasA_Cp orthologues (at the collision energy of 10 eV).

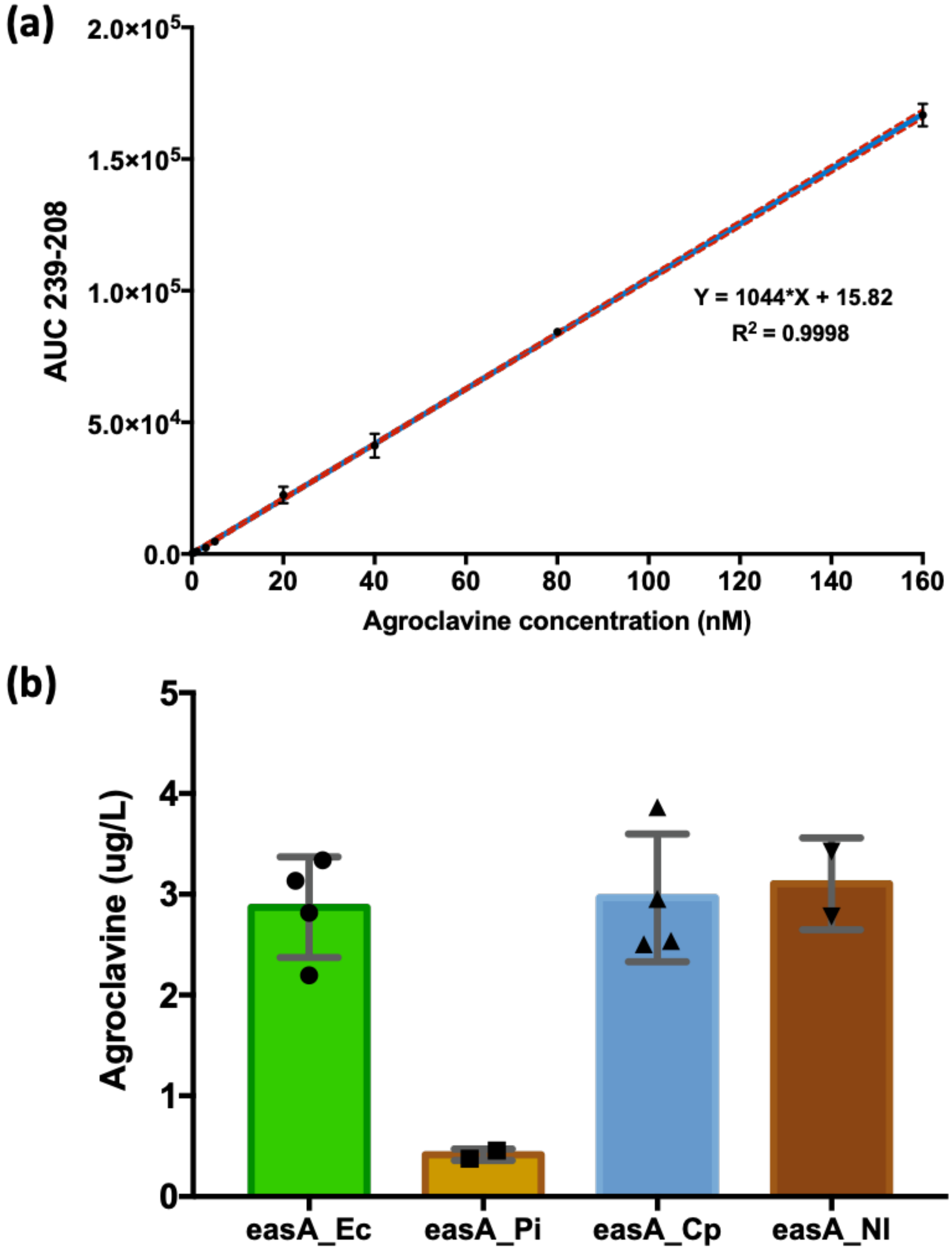

Supplementary Fig. 11. Quantification of agroclavine produced from the EasA screening strains. (a) Standard curve of agroclavine established in the negative control (YOCE). The curve was generated by plotting the peak area response for the ion transition of 239→ 208 m/z against spiked agroclavine concentrations. (b) Agroclavine titre from the screening strains calculated from the standard curve, measured by the peak area response for the ion transition of 239→ 208 m/z. Data are presented as mean values +/- standard deviation. Error bars representing standard deviations were calculated from three biological replicates.

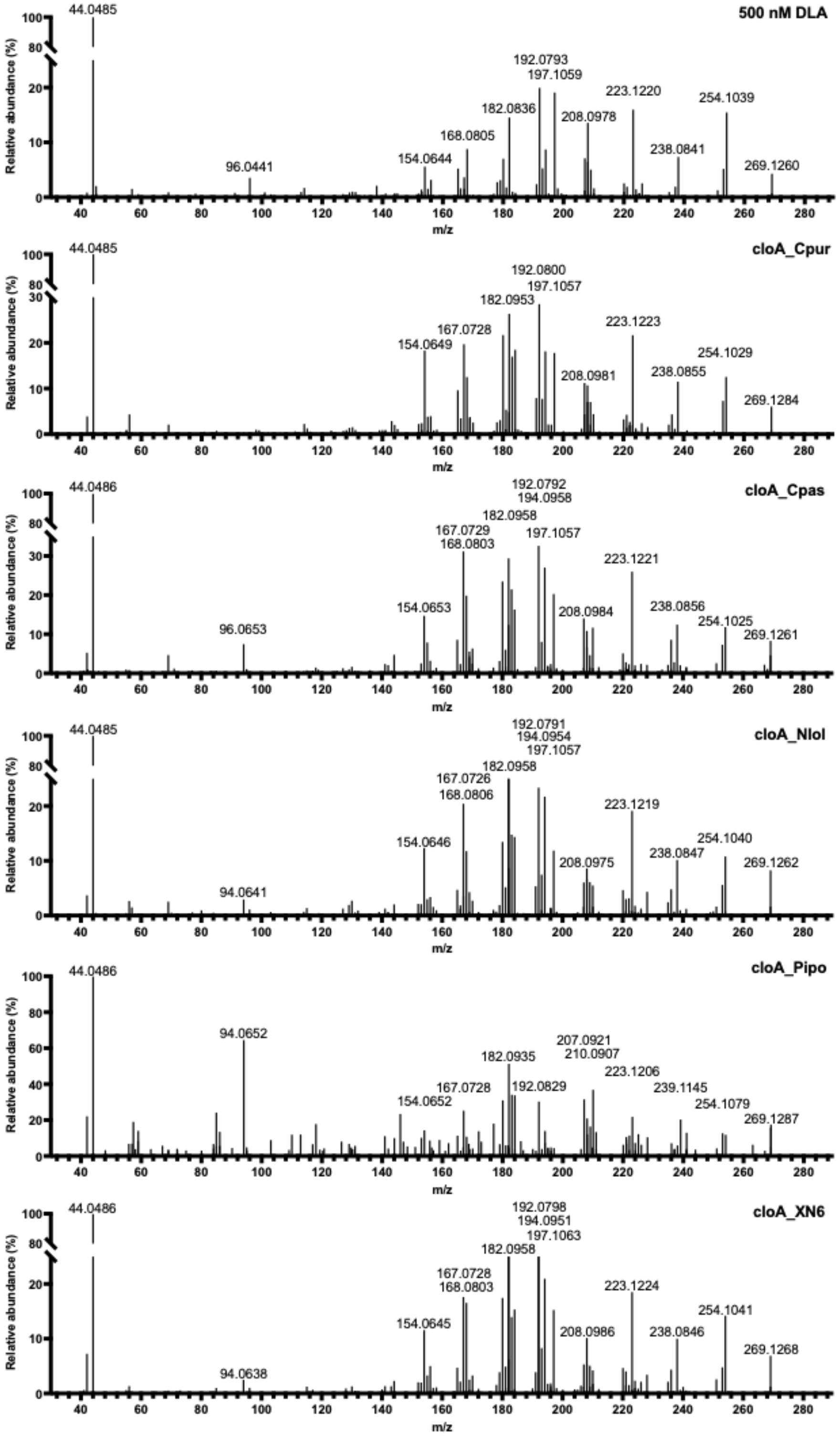

Supplementary Fig. 12. MS/MS fragmentation spectra of the peaks eluted at 126.3 seconds from the screen of CloA orthologs. Comparison of the fragmentation patterns against the commercial DLA standard against the compounds produced from the various CloA orthologs show the production of similar fragmentation ions (at the collision energy of 10 eV).

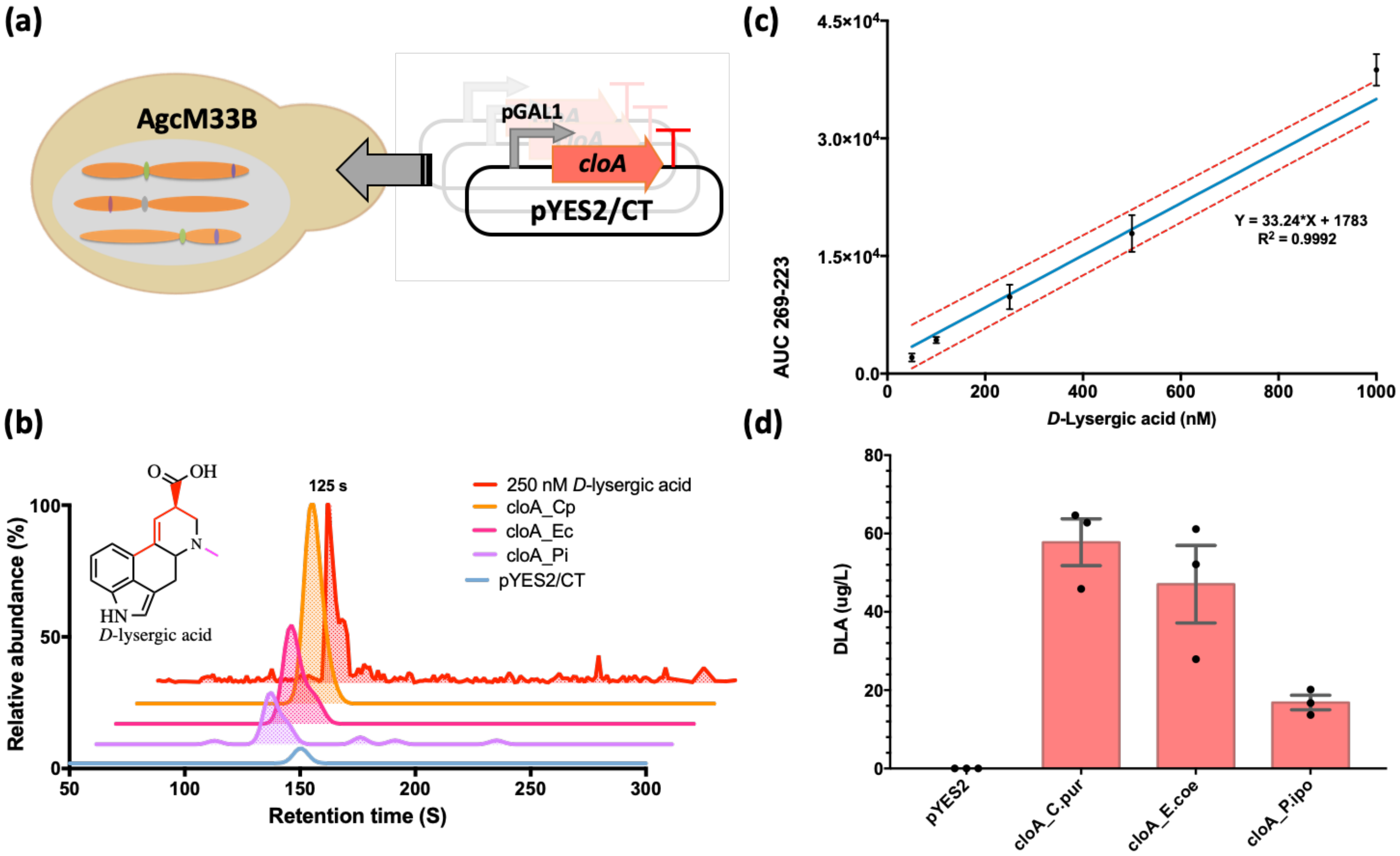

Supplementary Fig. 13. Assessing the performance selected CloA orthologues in the context of an agroclavine-producing yeast chassis. (a) Cartoon illustration of the experiment performed. (b) LC-MS/MS chromatograms of the products showing the ion transition of 269 → 223 m/z. (c) Standard curve of DLA spiked into samples of the empty vector control. The curve was obtained by plotting the peak area response for the ion transition of 269 → 223 m/z against spiked DLA concentration. (d) Quantification of DLA produced from AgcM33B strains expressing the CloA orthologues from an episomal plasmid. Data are presented as mean values +/- standard deviation. Error bars representing standard deviations were obtained from three biological replicates.

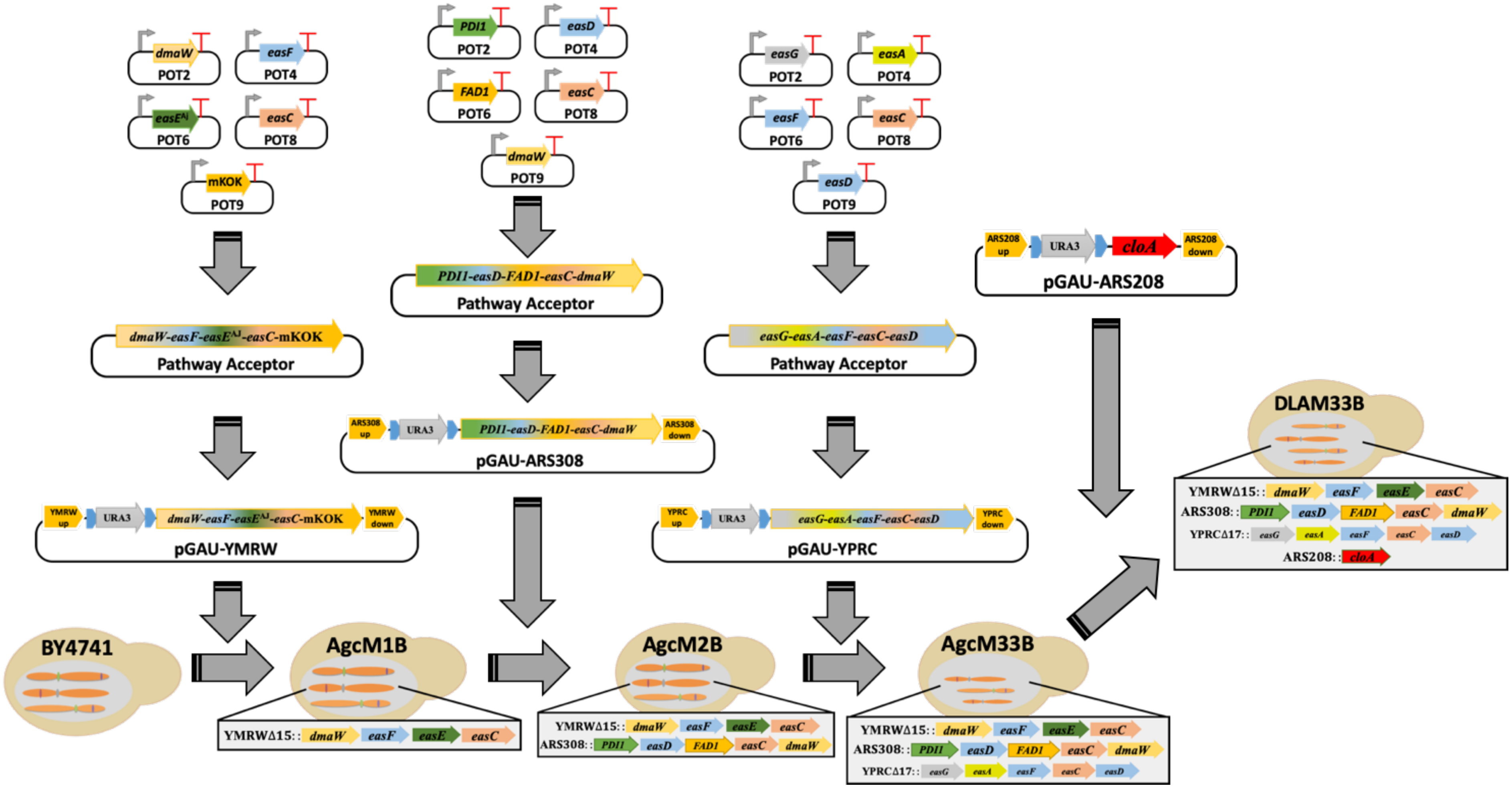

Supplementary Fig. 14. Schematic representation of the modular introduction of the four segments to sequentially reconstitute the pathway to *D*-lysergic acid. Following the modified YeastFab workflow, the genetic parts were condensed as transcriptional units on the POT plasmids before assembly as concatenated transcriptional units on the pathway acceptor vectors (p[C/M]K[U/L/H]). Verified pathway segments were then moved into the genome integration vectors (pGAU series). Integrated constructs were verified and cured of the URA3 selection marker for subsequent rounds of integration.

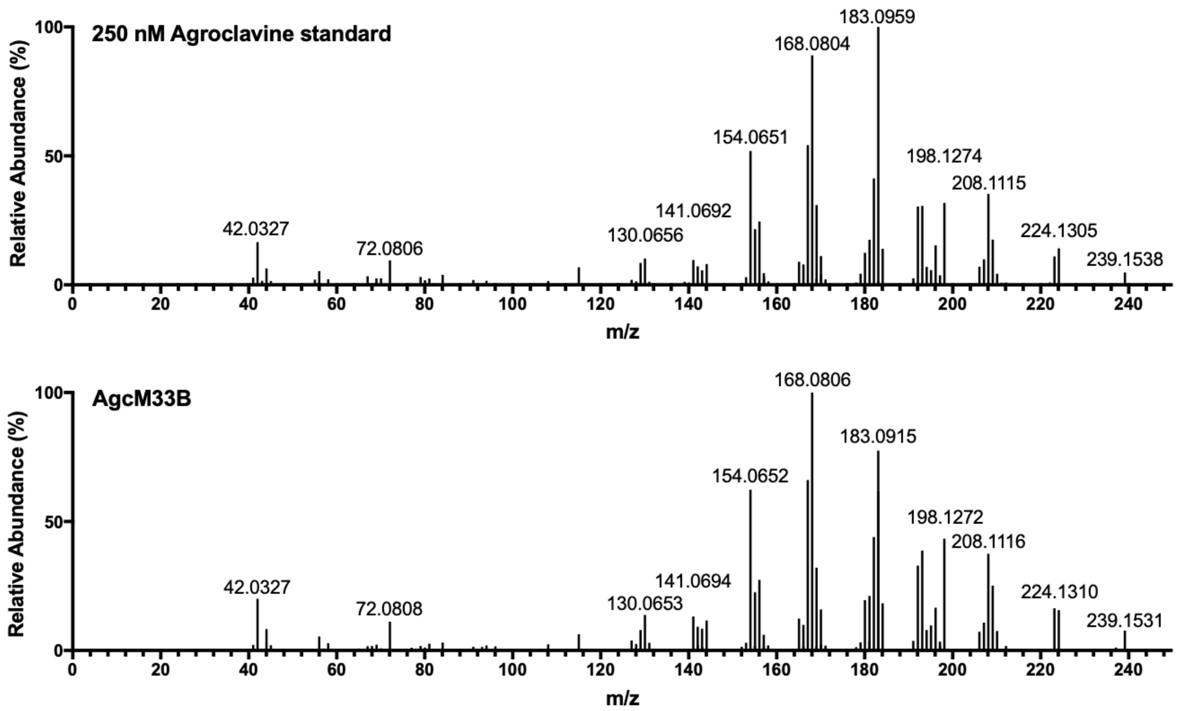

Supplementary Fig. 15. MS/MS fragmentation spectra of 250 nM agroclavine standard spiked in AgcM2B (top) and peak eluted from AgcM33B (bottom), demonstrating the production of agroclavine in the reconstituted strain. Fragmentation spectrum obtained at the collision energy of 20 eV.

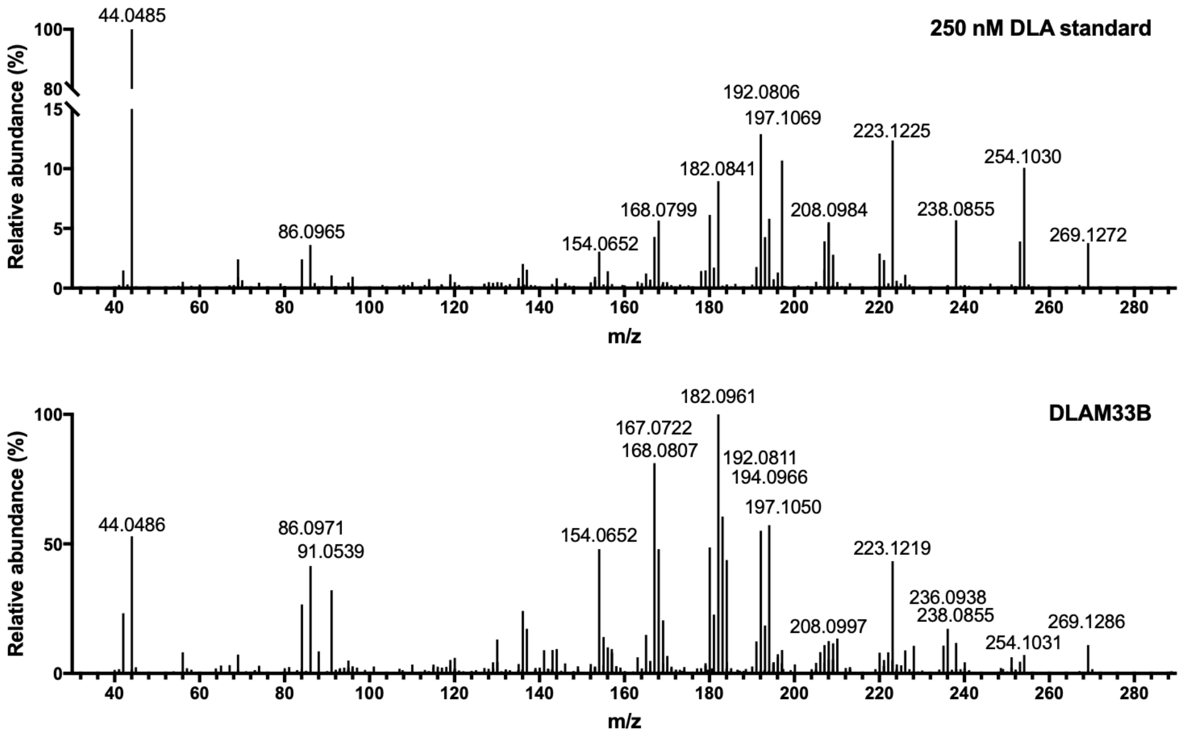

Supplementary Fig. 16. MS/MS fragmentation spectra of 250 nM DLA standard spiked in AgcM33B (top) and peak eluted from DLAM33B (bottom), demonstrating the production of DLA in the reconstituted strain. Fragmentation spectrum obtained at the collision energy of 20 eV.

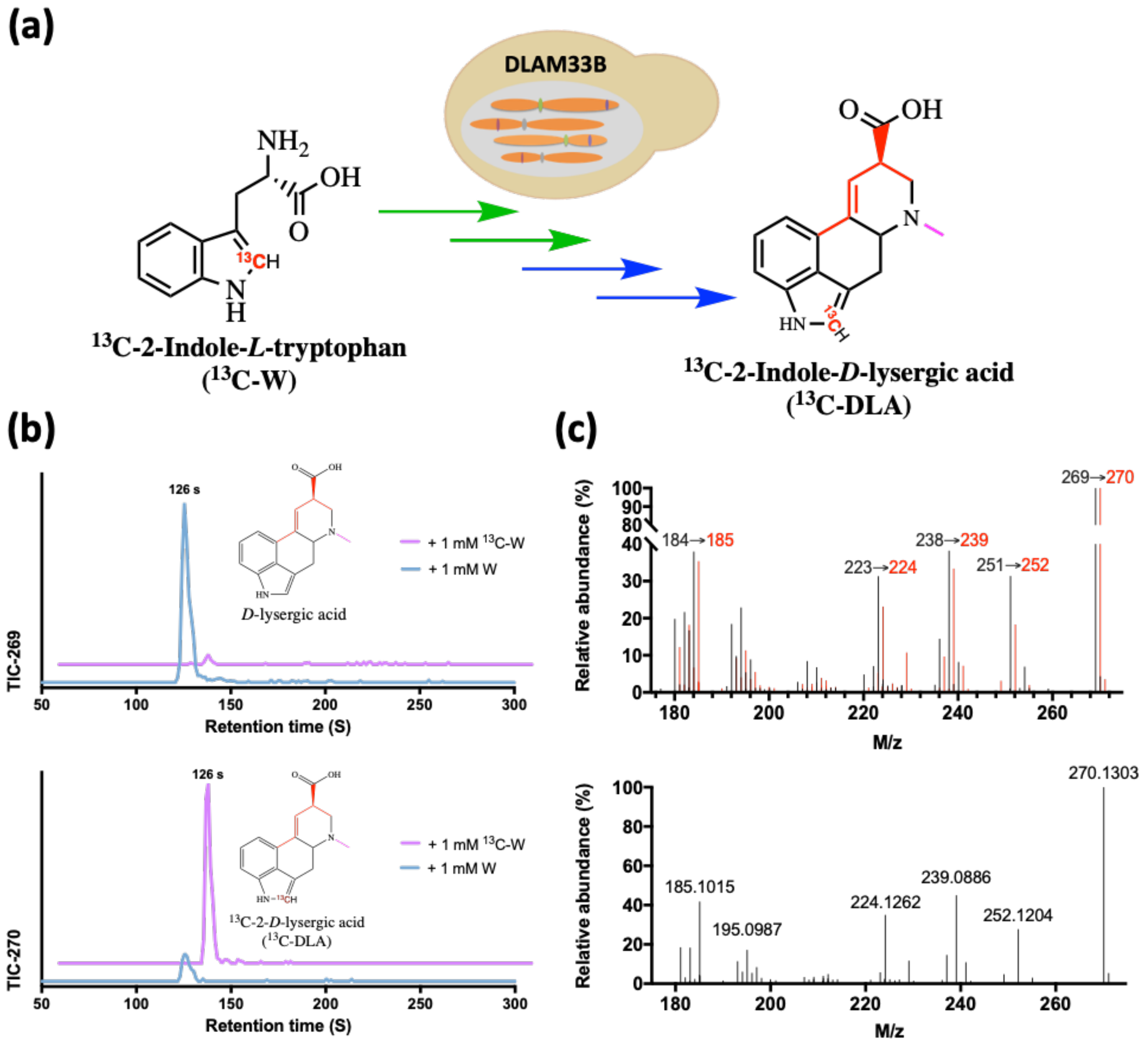

Supplementary Fig. 17. ^{13}C-tryptophan feedstock for DLAM33B confirms the production of DLA. (a) Schematic representation of the incorporation of ^{13}C-W by DLAM33B to produce ^{13}C-DLA. (b) LC-MS chromatograms showing the $[M+H]^+$ shift in the peak corresponding to the elution of DLA and ^{13}C-DLA. (c) (Top panel) Overlays of the MS/MS spectra obtained from samples supplied with tryptophan (black) and ^{13}C-W (red). (Bottom panel) MS/MS difference spectra between samples provided with tryptophan and ^{13}C-W, highlighting the +1 m/z shift for the $[M+H]^+$ expected for DLA and its fragmentation ions.

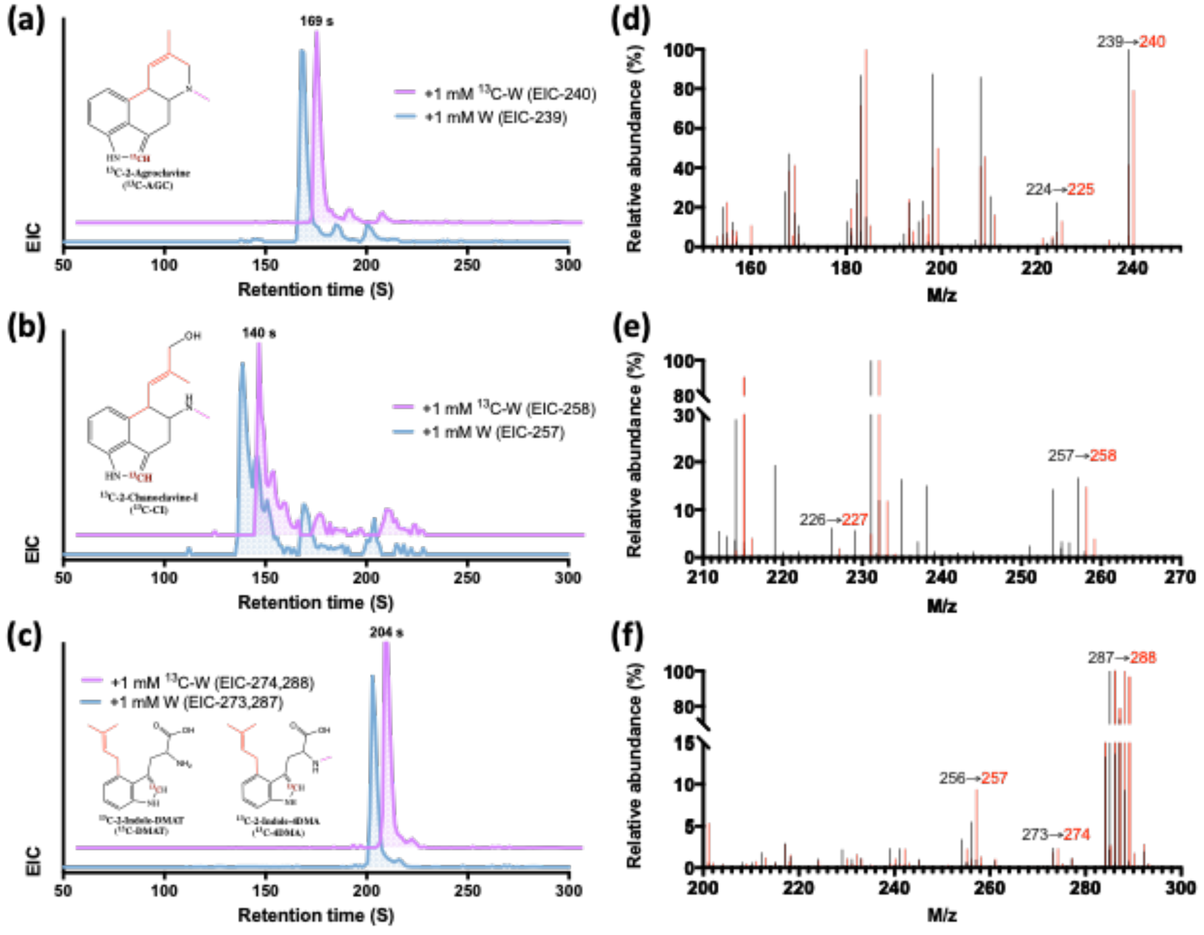

Supplementary Fig. 18. LC-MS chromatograms showing the incorporation of ^{13}C-labelled tryptophan in all intermediates along the ergot alkaloid biosynthesis pathway: (a) agroclavine (239 m/z) and ^{13}C-agroclavine (240 m/z); (b) chanoclavine-I (257 m/z) and ^{13}C-chanoclavine-I (258 m/z); (c) DMAT (273 m/z), 4DMA (287 m/z), ^{13}C-DMAT (274 m/z), and ^{13}C-4DMA (288 m/z). Overlaying the mass spectra between samples fed with ^{13}C-tryptophan (Red spectra) and tryptophan (Black spectra) highlight the +1 m/z shift for all precursor ions and their corresponding fragment ions (d, e, f).

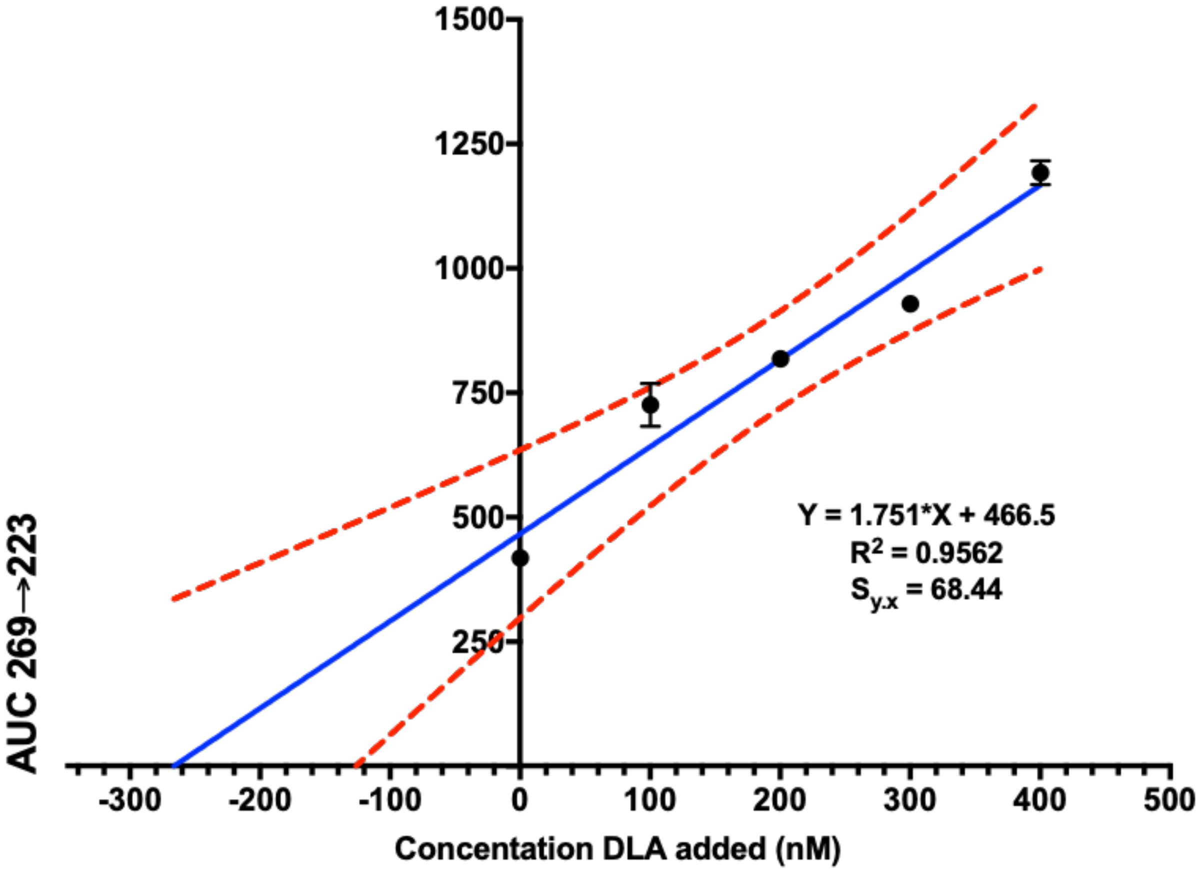

Supplementary Fig. 19. Quantification of DLA production titre from the DLAM33B strain by standard addition. The calibration curve was obtained by plotting the area under the curve for the peak response of the MS/MS transition of 269→223 m/z against the final concentration of DLA added to aliquots of the DLAM33B sample. Blue line represents the line of best fit across the points, dotted red lines represent the 95% confidence interval of the calculated line of best fit, Data are presented as mean values +/- standard deviation. Error bars of points were calculated from three biological replicates per calibration level.

SUPPLEMENTARY TABLES

Supplementary Table 1. List of promoter sequences characterized in this study.

Promoter name	Sequence
mdn1	CTTCCCATACTAAAGCTTTCTCTTGATATATAGTCAAACTGAATGTATACTGAAACAC<br>ATATTCAAGGCATTGATTTTAAATGCACTTGAACCGTTATTCCATTTCTTTTTCTACTC<br>ATCATTTATTGTCGCCCTTTACGAGGTATTTTTTCTTTTTCGAGTGAAAAATTTTAAAG<br>AAGCGATGAGCTAAGGTCTATTTAGCTTTATGAAAAGAAAACATTATATCACTTACT<br>GCTTTACTTTAGACATTCCTCTCCAAAGTGATTATTTTAAAGATTCAAAGTACCTCTG<br>AGTTAATTTGACATCGAATTCTACTCTTATTATTTTAAGGTACCTCTACTTTACGCGGT<br>CACATTATTTTTAAAACCGTTATAATCTGCTGAGA
vps13	CTGACACTATTGACAACGTTAAGTCGAAAATTCAAGACAAGGAAGGTATTCCTCCAG<br>ACCAGCAAAGATTGATTTTTGCCGGTAAGCAACTAGAAGATGGTAGAACGCTGTCGG<br>ACTACAATATTCAAAAGGAGTCCACTCTTCACCTTGTCTTGAGGTTGAGGGGTGGTA<br>ACTGATCAGTCCTCGCAATATTTTCATTATGTCAATATATATATGTTTACTCTCCTTTT<br>TTCTTTTTGGTTTTTTTTTTTTTTTGATAAATACTCCATAGAACACTAAATAAATTGTT<br>CAACTGTGTTATTGTCTTTATTCATGTTGGTTTTCAAGAGCTTGGATTTTTGAATCGTC<br>TTATACTATGACGTTCACTATTTTCGCGAAGCCGGGTAATACCATTAGCTATTTTGAT<br>AGAAAGGGATTTTTATTAGGGAATATAAACCACCTTTATAGTGTCCTATCATGTTTCA<br>ATCTCCAGTAAACGCACATAAGCCGACCAATTGAGTCAACCTTTTAACTCTATTTAAT<br>TTGATACGGATAGAATATTGTGACTACCAAAAGGGAAAAGGCAGAAAAAAGGAAAA<br>TTAAGAACAG
pgi1	AAAAGGTCCTTTCTTCATAATCAATGCTTTCTTTTACTTAATATTTTACTTGCATTCAG<br>TGAATTTTAATACATATTCCTCTAGTCTTGCAAAATCGATTTAGAATCAAGATACCAG<br>CCTAAAA
pma1	GTATTCTGATAAATCTAAAGAGAAATTACTAAAAAAAAGAAAAAAAAAAGAACGGG<br>GGTGTAATAATTTGTAGTTCATTATTGCAATTATATATCTATATCTATATATGTATATA<br>ACATTAACATGTGCATGTACACACGTAATCGCGCGTGTACATGTCTATATGTGTTACT<br>TGAACTATACTGTTTTGACGTGTATGTTTATTTATCTCTCTTCTGATTCCTCCACCCCT<br>TCCTTACTCAACCGGGTAAATGTCGCATCATGACTCCCGACAATAATCCCCTCTGGTA<br>TAGCGAGAAGCAACTTTAGCTTCTTAACGGCAAGAACTTTTTTATGTTTGTCGCACCT<br>GTATCTTCACAAAAGTTGGATACAGCAATAAGAAAGGAAACCACATTTGTGCCA
tra1	CACCTTCAATAGGGCAAACAGAAGGACGAGCTGCATCACTTTTAGCCGCCACTAATC<br>AACTCCTTCAGATAGATCGCCAGAACGCAAATAAGTGAAATTTGAAGATCAACTTGA<br>ATCAAAAAGCCAAAACTAAGAATTGTACGCTACTCTGCCAGACAATATTTTACGGAC<br>ACGCGCATTTCAAGACAAAGCCATAATTCATGCCTTTTCAACTAGCATCTGGACGCC<br>GCTGAGATCATAGGAACAAACAAAACGCACGAAAATAGCTAACGCACGTATCGAAT<br>ACCAAATCAGTCTGCATTTATTGTACCCTCAAATTACAAATTCAACCAACAATTGTTC<br>AAAAGACCACCTTGAATCTTGTGTTTCCTAATTTGGTAAGTGATCACGTGGTCAGACA<br>GTTTTCCAGGTCAAACTGAAAAAAAAGCTCAAGAACCATTTTTCTTTAATAGCCTTTT<br>TTAGTTGTATTCTAATAAACAACACCAACAAAATCGGAGGTATCACACGTAATTACA<br>TAAAGAAGATTAAATTCAGAGAAGATCTATCACACGATATCTCCATAGTTACAAAGA<br>ATACCGCATTTTGCCG

dyn1	AATCAGTCGGAAATGTTCCTTGGACATAAGATATCAGCAGGCACGTCTTCCCTACAG<br>CGCCATCTCCCACAACAACAATCTTTAGGTGATAATCTGGCAGTTTGTTTGTACGTTT<br>CATCTGCTCATAACTAGGAATGGTAGAAAGGCTTCTAGCGAATGGAGTAGGCAATCT<br>GGGAAGATTCTTTTCATCTAAAGTGCCAGGTGATTCAGGAAGATTGCTACTTGAGGG<br>CGATCCCTGCGAAACTATGTTACTTTCGTTCCCACAATTGCCACCTTTTCGCTTAAAT<br>AATAGTGTATTCATGAAAAAGCGTTACTATTGGAGATTTTTGGACCTATTTGACTTAA<br>GTTTGCTGTAACTTTTGAGGGATGCTTATCATGTCTTTTGTCGTCTTAACGTAAGGCTA<br>TAACTACTTAATCAGATCATCCCGAATTGATGCGCGGAAGGTCGCATTGTACGGATT<br>CTAATAAATAAGCTAATGAAAGGTAAACAAACATTACATATAAAAATTAACAGAAA<br>GCATAGAACATAGAAGGGCAATAATACTCGTTCAGAGCTTAAATTGGAAAGTACGTC<br>AAAACGTTTTTTAGGC
rpl15a	TCGACACTTACTTAATATGTTTTGCCGCCCTTCATAAGAGGGTGTTTCTAAAATTTATT<br>GGGCAAGAATGAGATGGACTCGCACCCTACATGACGTTTAAATATTTAGTGTTAAGG<br>TTCAGAACATGCACCAGGTGCGACATGTGTTGCGATTATCATGACAATGTCTCTATCC<br>GAGATGCATTTGTAGTATCAATTGATGCGTATTATGACATGATTTACATAGCATACAT<br>CGTCAAACATGATATTATATTCTTTTTTTGATAAATGTACGGATTTAAAGCTGTCGAA<br>TATATTTTCTGAAATTTCTTGGAGCTGACGCAAAATTTTCAAAGGTGCTAAAATTTTT<br>CAAGATTTCTCACTTTTGCTTGGTAACAAAGAATGATGGCATTGCATTTTTACCACCG<br>GTACATTTAACTGCTATTTCTCACGTTTCTTTCCCTATCCTTAAGTAATTCTTTTACAA<br>TCTAAGAAAACCACGATCAAACAAATAAATCAGCA
rpl8b	GGCAAAGAGGGATAGCTTCACTGCCAATCACTACACTCTGTTCTGAAGGAGGGGAAT<br>TACTGTTGTCTCATAAAAAAACACCTTTGAAACACTTTCACTCATAGAGAAGCCAAG<br>AACTCTGATGAGTTCTTTATTTGTTGCCGCTAACTTTATCTTATCTTCTCCATTGTAAT<br>AATTACTATTCTTTTTACTTGCCTGTTTTCCATCCCAATGAAGATCCGTACATCTTAGA<br>CACCCTTTTCTTTTTTTCTTTTTTTTTTTTTTTTTTTTTTTTTGGCTTCCGAAGTGCCGACA<br>ACTAAAGAATTATCGCATTTTTTTTGAATACATCATACTATACGATAGAACGCATTGA<br>AACTTTTCCCATCTCAAAATCCAGGGACAATAGTATGGGATCGATATTATTATCAAG<br>ACATAAGTATTGTGATGCGCACGTTGAAATTCACTATTTTTTTTTTTTATATCTGTTTT<br>TCAATACAGTGAACACGATAACTGAAAGAAAAG
ssa1	CAGATTTTAGTTGAGAAAGTAATTAAATTATTCTTCTTTTTCCAGAACGTTCCATCGG<br>CGGCAAAAGGGAGAGAAAGAACCCAAAAAGAAGGGGGGCCATTTAGATTAGCTGAT<br>CGTTTCGAGGACTTCAAGGTTATATAAGGGGTGGATTGATGTATCTTCGAGAAGGGA<br>TTGAGTTGTAGTTTCGTTTCCCAATTCTTACTTAAGTTGTTTTATTTTCTCTATTTGTAA<br>GATAAGCACATCAAAAGAAAAGTAATCAAGTATTACAAGAAACAAAAATTCAAGTA<br>AATAACAGATAAT
tdh2	TAATTCAATAAGTATGTCATGAAATACGTTGTGAAGAGCATCCAGAAATAATGAAAA<br>GAAACAACGAAACTGGGTCGGCCTGTTGTTTCTTTTCTTTACCACGTGATCTGCGGCA<br>TTTACAGGAAGTCGCGCGTTTTGCGCAGTTGTTGCAACGCAGCTACGGCTAACAAAG<br>CCTAGTGGAACTCGACTGATGTGTTAGGGCCTAAAACTGGTGGTGACAGCTGAAGTG<br>AACTATTCAATCCAATCATGTCATGGCTGTCACAAAGACCTTGCGGACCGCACGTAC<br>GAACACATACGTATGCTAATATGTGTTTTGATAGTACCCAGTGATCGCAGACCTGCA<br>ATTTTTTTGTAGGTTTGGAAGAATATATAAAGGTTGCACTCATTCAAGATAGTTTTTT<br>TCTTGTGTGTCTATTCATTTTATTATTGTTTGTTTAAATGTTAAAAAAACCAAGAACTT<br>AGTTTCAAATTAAATTCATCACACAAACAAACAAAACAAA
pyk1	ACAGATTGGGAGATTTTCATAGTAGAATTCAGCATGATAGCTACGTAAATGTGTTCC<br>GCACCGTCACAAAGTGTTTTCTACTGTTCTTTCTTCTTTCGTTCATTCAGTTGAGTTGA<br>GTGAGTGCTTTGTTCAATGGATCTTAGCTAAAATGCATATTTTTTCTCTTGGTAAATG<br>AATGCTTGTGATGTCTTCCAAGTGATTTCCTTTCCTTCCCATATGATGCTAGGTACCTT<br>TAGTGTCTTCCTAAAAAAAAAAAAAAGGCTCGCCATCAAAACGATATTCGTTGGCTTT<br>TTTTTCTGAATTATAAATACTCTTTGGTAACTTTTCATTTCCAAGAACCTCTTTTTTCC<br>AGTTATATCATGGTCCCCTTTCAAAGTTATTCTCTACTCTTTTTCATATTCATTCTTTTT<br>CATCCTTTGGTTTTTTATTCTTAACTTGTTTATTATTCTCTCTTGTTTCTATTTACAAGA<br>CACCAATCAAAACAAATAAAACATCATCACA

cup1	GCCTTGTTACTAGTTAGAAAAAGACATTTTTGCTGTCAGTCACTGTCAAGAGATTCTT<br>TTGCTGGCATTTCTTCTAGAAGCAAAAAGAGCGATGCGTCTTTTCCGCTGAACCGTTC<br>CAGCAAAAAAGACTACCAACGCAATATGGATTGTCAGAATCATATAAAAGAGAAGC<br>AAATAACTCCTTGTCTTGTATCAATTGCATTATAATATCTTCTTGTTAGTGCAATATCA<br>TATAGAAGTCATC
pdc1	TTATGTATGCTCTTCTGACTTTTCGTGTGATGAGGCTCGTGGAAAAAATGAATAATTT<br>ATGAATTTGAGAACAATTTTGTGTTGTTACGGTATTTTACTATGGAATAATCAATCAA<br>TTGAGGATTTTATGCAAATATCGTTTGAATATTTTTCCGACCCTTTGAGTACTTTTCTT<br>CATAATTGCATAATATTGTCCGCTGCCCCTTTTTCTGTTAGACGGTGTCTTGATCTACT<br>TGCTATCGTTCAACACCACCTTATTTTCTAACTATTTTTTTTTTAGCTCATTTGAATCA<br>GCTTATGGTGATGGCACATTTTTGCATAAACCTAGCTGTCCTCGTTGAACATAGGAAA<br>AAAAAATATATAAACAAGGCTCTTTCACTCTCCTTGCAATCAGATTTGGGTTTGTTCC<br>CTTTATTTTCATATTTCTTGTCATATTCCTTTCTCAATTATTATTTTCTACTCATAACCT<br>CACGCAAAATAACACAGTCAAATCAATCAAA
pda1	GTATTCTGATAAATCTAAAGAGAAATTACTAAAAAAAAGAAAAAAAAAAAGAACGGG<br>GGTGTAATAATTTGTAGTTCATTATTGCAATTATATATCTATATCTATATATGTATATA<br>ACATTAACATGTGCATGTACACACGTAATCGCGCGTGTACATGTCTATATGTGTTACT<br>TGAACTATACTGTTTTGACGTGTATGTTTATTTATCTCTCTTCTGATTCCTCCACCCCT<br>TCCTTACTCAACCGGGTAAATGTCGCATCATGACTCCCGACAATAATCCCCTCTGGTA<br>TAGCGAGAAGCAACTTTAGCTTCTTAACGGCAAGAACTTTTTATGTTTGTCGCACCT<br>GTATCTTCACAAAAGTTGGATACAGCAATAAGAAAGGAAACCACATTTGTGCCA
cyc1	AATTTTTTTGGAAAACCAAGAAATGAATTATATTTCCGTGTGAGACGACATCGTCGA<br>ATATGATTCAGGGTAACAGTATTGATGTAATCAATTTCCTACCTGAATCTAAAATTCC<br>CGGGAGCAAGATCAAGATGTTTTCACCGATCTTTCCGGTCTCTTTGGCCGGGGTTTAC<br>GGACGATGGCAGAAGACCAAAGCGCCAGTTCATTTGGCGAGCGTTGGTTGGTGGATC<br>AAGCCCACGCGTAGGCAATCCTCGAGCAGATCCGCCAGGCGTGTATATATAGCGTGG<br>ATGGCCAGGCAACTTTAGTGCTGACACATACAGGCATATATATATGTGTGCGACGAC<br>ACATGATCATATGGCATGCATGTGCTCTGTATGTATATAAAACTCTTGTTTTCTTCTTT<br>TCTCTAAATATTCTTTCCTTATACATTAGGACCTTTGCAGCATAAATTACTATACTTCT<br>ATAGACACACAAACACAAATACACACACTAAATTAATA
tpi1	ATTTAAACTGTGAGGACCTTAATACATTCAGACACTTCTGCGGTATCACCCTACTTAT<br>TCCCTTCGAGATTATATCTAGGAACCCATCAGGTTGGTGGAAGATTACCCGTTCTAAG<br>ACTTTTCAGCTTCCTCTATTGATGTTACACCTGGACACCCCTTTTCTGGCATCCAGTTT<br>TTAATCTTCAGTGGCATGTGAGATTCTCCGAAATTAATTAAAGCAATCACACAATTCT<br>CTCGGATACCACCTCGGTTGAAACTGACAGGTGGTTTGTTACGCATGCTAATGCAAA<br>GGAGCCTATATACCTTTGGCTCGGCTGCTGTAACAGGGAATATAAAGGGCAGCATAA<br>TTTAGGAGTTTAGTGAACTTGCAACATTTACTATTTTCCCTTCTTACGTAAATATTTTT<br>CTTTTTAATTCTAAATCAATCTTTTTCAATTTTTTGTTTGTATTCTTTTCTTGCTTAAAT<br>CTATAACTACAAAAAACACATACATAAACTAAAA
tdh3	ACAGTTTATTCCTGGCATCCACTAAATATAATGGAGCCCGCTTTTTAAGCTGGCATCC<br>AGAAAAAAAAAGAATCCCAGCACCAAAATATTGTTTTCTTCACCAACCATCAGTTCA<br>TAGGTCCATTCTCTTAGCGCAACTACAGAGAACAGGGGCACAAACAGGCAAAAAAC<br>GGGCACAACCTCAATGGAGTGATGCAACCTGCCTGGAGTAAATGATGACACAAGGC<br>AATTGACCCACGCATGTATCTATCTCATTTTCTTACACCTTCTATTACCTTCTGCTCTC<br>TCTGATTTGGAAAAAGCTGAAAAAAAAGGTTGAAACCAGTTCCCTGAAATTATTCCC<br>CTACTTGACTAATAAGTATATAAAGACGGTAGGTATTGATTGTAATTCTGTAAATCTA<br>TTTCTTAAACTTCTTAAATTCTACTTTTATAGTTAGTCTTTTTTTTAGTTTTAAAACACC<br>AAGAACTTAGTTTCGAATAAACACACATAAACAAACAAA

eno2	CGACGTGCACCAACTTGCGGAAAGTGGAATCCCGTTCCAAAACTGGCATCCACTAAT<br>TGATACATCTACACACCGCACGCCTTTTTTCTGAAGCCCACTTTCGTGGACTTTGCCA<br>TATGCAAAATTCATGAAGTGTGATACCAAGTCAGCATACACCTCACTAGGGTAGTTT<br>CTTTGGTTGTATTGATCATTTGGTTCATCGTGGTTCATTAATTTTTTTTCTCCATTGCTT<br>TCTGGCTTTGATCTTACTATCATTTGGATTTTTGTCGAAGGTTGTAGAATTGTATGTGA<br>CAAGTGGCACCAAGCATATATAAAAAAAAAAAGCATTATCTTCCTACCAGAGTTGAT<br>TGTTAAAAACGTATTTATAGCAAACGCAATTGTAATTAATTCTTATTTTGTATCTTTTC<br>TTCCCTTGTCTCAATCTTTTATTTTTATTTTATTTTTCTTTTCTTAGTTTCTTTCATAACA<br>CCAAGCAACTAATACTATAACATACAATAATA
pgk1	TGTTTGCAAAAAGAACAAAACTGAAAAAACCCAGACACGCTCGACTTCCTGTCTTCC<br>TATTGATTGCAGCTTCCAATTTCGTCACACAACAAGGTCCTAGCGACGGCTCACAGGT<br>TTTGTAACAAGCAATCGAAGGTTCTGGAATGGCGGGAAAGGGTTTAGTACCACATGC<br>TATGATGCCCACTGTGATCTCCAGAGCAAAGTTCGTTCGATCGTACTGTTACTCTCTC<br>TCTTTCAAACAGAATTGTCCGAATCGTGTGACAACAACAGCCTGTTCTCACACACTCT<br>TTTCTTCTAACCAAGGGGGTGGTTTAGTTTAGTAGAACCTCGTGAAACTTACATTTAC<br>ATATATATAAACTTGCATAAATTGGTCAATGCAAGAAATACATATTTGGTCTTTTCTA<br>ATTCGTAGTTTTTCAAGTTCTTAGATGCTTTCTTTTTCTCTTTTTTACAGATCATCAAG<br>GAAGTAATTATCTACTTTTTACAACAAATATAAAACA
yef3	ATTAAAAAAACAACTTACAATCATTGTTCGCCCCTTCCATACTTACTGCCACTCGCAA<br>AAGGGCCCAACCAGGGCAATTACGTATCAAAAAATCATGACAGGCTGGGTAATAAA<br>TATTCGTGAAGAAAGAAGAAATTAAAAAAAGAAACGAAGAAGCAAAAAAAAGAAA<br>AGACTCCGTTTAATCACTTTCAACCGCGGTTTATCCGGCCCCACCCATGCATAACCCT<br>AAATTATTAGATCACTTAGCACGTGAAAAAGAAACGTTTTTAATGTTTTTTTTTTTTTT<br>TTCTTTTTCTTTTTTTGCGTTGGTGAAAATTTTTTCGCTTCCTCGAGTATAATTATCTCA<br>TCTCATCTTTCATATAAGATAAGAAGTTTATAAAAACCTTTTGCATCAAAATTTTGT<br>AGAATATCTCTTTTTCTTACGCTCTCTTTCTTTCCTTAATTGTTTTCTAAAGAACCGTG<br>TATTTTTCTAGTTCGAATCCATCGATAACATTAAAAG
tef2	GGGCGCCATAACCAAGGTATCTATAGACCGCCAATCAGCAAACTACCTCCGTACATT<br>CATGTTGCACCCACACATTTATACACCCAGACCGCGACAAATTACCCATAAGGTTGTT<br>TGTGACGGCGTCGTACAAGAGAACGTGGGAACTTTTTAGGCTCACCAAAAAAGAAA<br>GAAAAAATACGAGTTGCTGACAGAAGCCTCAAGAAAAAAAAAAATTCTTCTTCGACTA<br>TGCTGGAGGCAGAGATGATCGAGCCGGTAGTTAACTATATATAGCTAAATTGGTTCC<br>ATCACCTTCTTTTCTGGTGTCGCTCCTTCTAGTGCTATTTCTGGCTTTTCCTATTTTTTT<br>TTTTCCATTTTTCTTTCTCTCTTTCTAATATATAAATTCTCTTGCATTTTCTATTTTTCTC<br>TCTATCTATTCTACTTGTTTATTCCCTTCAAGGTTTTTTTTTAAGGAGTACTTGTTTTTA<br>GAATATACGGTCAACGAACTATAATTAACTAAAC
rpl3	CCGGACAGTAATATAGTAATCGTTTTGTACGTTTTTCAAGAAGCGACGCACAACTGTT<br>TTCCATTTTTTTTTTTTTTTTTTTCAGTGATCATCGTCCATGAAAAAAATTTTTCATTTGT<br>CTCTTTCGTGCTTCCTGGATATATAAAATACGATTTATTTAGTTGTCTTTGTCAATCCT<br>CATCTTTCTTTACTCATTATTTCATTTCGGTTTTGTCATCTCTAGAACAACACAGTTAC<br>TACAACAATCAATC
act1	TAACCTACATTCTTCCTTATCGGATCCTCAAAACCCTTAAAAACATATGCCTCACCCT<br>AACATATTTTCCAATTAACCCTCAATATTTCTCTGTCACCCGGCCTCTATTTTCCATTT<br>TCTTCTTTACCCGCCACGCGTTTTTTTCTTTCAAATTTTTTTCTTCCTTCTTCTTTTTCTT<br>CCACGTCCTCTTGCATAAATAAATAAACCGTTTTGAAACCAAACTCGCCTCTCTCTCT<br>CCTTTTTGAAATATTTTTGGGTTTGTTTGATCCTTTCCTTCCCAATCTCTCTTGTTTAAT<br>ATATATTCATTTATATCACGCTCTCTTTTTATCTTCCTTTTTTTCCTCTCTCTTGTATTCT<br>TCCTTCCCCTTTCTACTCAAACCAAGAAGAAAAAGAAAAGGTCAATCTTTGTTAAAG<br>AATAGGATCTTCTACTACATCAGCTTTTAGATTTTTCACGCTTACTGCTTTTTTCTTCC<br>CAAGATCGAAAATTTACTGAATTAACA

gpm1	GTGATGTCTAAGTAACCTTTATGGTATATTTCTTAATGTGGAAAGATACTAGCGCGCG<br>CACCCACACACAAGCTTCGTCTTTTCTTGAAGAAAAGAGGAAGCTCGCTAAATGGGA<br>TTCCACTTTCCGTTCCCTGCCAGCTGATGGAAAAAGGTTAGTGGAACGATGAAGAAT<br>AAAAAGAGAGATCCACTGAGGTGAAATTTCAGCTGACAGCGAGTTTCATGATCGTGA<br>TGAACAATGGTAACGAGTTGTGGCTGTTGCCAGGGAGGGTGGTTCTCAACTTTTAAT<br>GTATGGCCAAATCGCTACTTGGGTTTGTTATATAACAAAGAAGAAATAATGAACTGA<br>TTCTCTTCCTCCTTCTTGTCCTTTCTTAATTCTGTTGTAATTACCTTCCTTTGTAATTTTT<br>TTTGTAATTATTCTTCTTAATAATCCAAACAAACACACATATTACAATA
fec-3	CCTCCTTGAAACTGAAATTTTAGCATGTGATTAATTAACTTGTAATATTCTAATCAAG<br>CTTATAAAAGAACGATCTACCGACTGTTTCGCAGAGGGCCAAAAAAGCATCGAAAA<br>AA
fec-1	CCTCCTTGAAACTGAAATTTTAGCATGTGATTAATTAACTTGTAATATTCTAATCAAG<br>CTTATAAAAGAGCACTGTTGGGCGTGAGTGGAGGCGCCGGAAAAAAGCATCGAAAA<br>AA
bda-1	GCTCAACGGCACAGAGGGGCGGGGGCGGTGTTAATTAACTTGTAATATTCTAATCAA<br>GCTTATAAAAGAGCACTGTTGGGCGTGAGTGGAGGCGCCGGAAAAAAGCATCGAAA<br>AAA
bda-3	GCTCAACGGCACAGAGGGGCGGGGGCGGTGTTAATTAACTTGTAATATTCTAATCAA<br>GCTTATAAAAGAACGATCTACCGACTGTTTCGCAGAGGGCCAAAAAAGCATCGAAA<br>AAA

Supplementary Table 2. List of terminator sequences used in this study.

Terminator name	Sequence
apl2	CTATAAACGTCCGTTGTAGTGAACTTAAGTATTATACCATACATATATACCCTTC<br>GTATTAATTTTTCCTTTTTATTGTTGGGCTGTGTGTATATAATTTATGTACAAGAA<br>TGGATATATCAAAAAATCCTTAAAAGATGTAAATCAAAAACATCAATCAACACA<br>GCTTATTTTGTAGAGCTTTGTTTTTGACATAACTTTTCAAGCTTGTTGATTTTTTCT<br>AAAACAATTTTATCCCTTTCACCATCTAAATATTTCTCATCTGTTTGATAAAACAG<br>GAGGCACGATTTCATTTCATCTCGTTGCTTATCACAAGTGGGAAAAACTTTCATT<br>CCATTTGCCTTAGCACAATCTGCCATTGCTTGAACAAAATTTGCACATTTTTTGTA<br>TGTTTCCGTCTTCAAGTTCTTGCGAGCCTGTTGT
atg10	GGAAAGCTTTTTAAAACACTATGCAATCATCTATGTAAATATATATATATATATA<br>TATTATACCGGCGGTAGTAAGCAGTATTTTTTTCTATCAGTCTATATTATTTGTTT<br>CTTTTTCTTGTCTGTTTTCTCATGTTACCTTCTTAGCATTACCATAATGCGAATCTT<br>CTGATGATGTGTCGGATAACCCAATAGAATCAGTCGTCGTCGCACCTAGCGAAG<br>CAGTTTCAATGGAATCTTCTTCTTCAAGAATTGTTGGTATACCACCAAACGTTCTT<br>TTGCCGCGCTGAACAGATTTAAATTGCACTTTCTTTTCTCCTGCTTCATTTTCGTT<br>GTCTGAAATAGATGACTTGGTCTTTGTCTTTTGAAGAGCCATTCTTTCTCCAAATT<br>CCTCATCATCATCGTTATTGTATGAGAAGTCACTCAGGTCCGATGTTCTTCTTGCT<br>CTATTTCTCTTCTTCCAACCAGGCCTTCTAAACCA
bna4	AGCCAGTTTATTCTTGCCATCCGTGTACGCTAGGAGAGGATTATTAAAATAAGTG<br>ATATATACATATATATATATATATATATATATATAATACACTAATTATTTTATGTG<br>ATGTTGATCACGCGAAACGGTAAACGGCTCTGTTCGCGCTTTCTTTGTTTACATTT<br>TAGTGAAGTATTGTCAAGATAATATCCATATGGTTGAACTTTTTATATTGGCTAG<br>TTAAATACTCTATTTATTGCACCAAAAAATCATCTTAGTGGACTTTTTGGAGCAA<br>AAAAAACCAGCAGGTAGTTATATAAGATGACTACACAACTAAGGTATGAAAATA<br>ACGATGACGATGAAAGAGTAGAATATAATCTCTTTACCAATAGATCCACCATGA<br>TGGCAAATTTTGAAGAATGGATCAAAATGGC
bud32	TATAAATGCTAGCGTACTGCCTGTATATCGCTGCACATAATATGTATCTATAATTT<br>ATATTAACGTACATAACTTTTAGGTATGTTACCATCAAAGCGTGTCCGAGTTATT<br>GAGATTTTGACAAATTCAGACAAATCATTTTTGAAGAACCATATATATAAATATA<br>TAAAGAAAAGAAAGGTAAACCAAAGACAGCAAACAATCGAAAAGTGGACAACC<br>AGCAATGGTAGATTCAATTCACCGTATTGCATCGGCGTTGGATACGGCCAAAGTA<br>ATAACGAGGGAAGCTGCCGCGGTAGCCACCTCTAAGCTGGGTGAATCCTCCTAT<br>ACTTATTATTCTCAAAACATCAATCCTCAACAGTTAGTCACCTTGTTAAATTCTAG<br>GAACTCTAGAGAAGTTAGAGATGCAATGAAGAGAATAATATCTATAATGGCTTC<br>CGATGATGACTC
cbr1	GATAAGGAACTGTAACAGAGTGCCATATATATATATATAGATTGGAACATATAT<br>AATATATACGCTATTTAGTTTAGTCACCTAAACGCACCGTTTCCATATTTCGTGCT<br>GGACGATTTTCGACTCGATAATACGTATAAGATCGGTACTAATAGTAACAAAAG<br>TAAAGGTAATACTTGCTTCAACTGTACAAAGGAGCATAGAACGTGGGGCAATTT<br>ACAAGCGATGTCGACGTCAGTCCCAGTCAAGAAGGCATTAAGTGCTCTTTTACGC<br>GATCCAGGAAACAGTCATTGTGCCGACTGTAAGGCGCAACTACATCCACGCTGG<br>GCTTCCTGGTCACTTGGTGTTTTCATTTGCATTAAATGTGCTGGTATACATAGATC<br>ATTGGGAACGCACATTTCGAAAGT

cyc1	ACAGGCCCCTTTTCCTTTGTCGATATCATGTAATTAGTTATGTCACGCTTACATTC<br>ACGCCCTCCTCCCACATCCGCTCTAACCGAAAAGGAAGGAGTTAGACAACCTGA<br>AGTCTAGGTCCCTATTTATTTTTTTTAATAGTTATGTTAGTATTAAGAACGTTATT<br>TATATTTCAAATTTTTCTTTTTTTTCTGTACAAACGCGTGTACGCATGTAACATTA<br>TACTGAAAACCTTGCTTGAGAAGGTTTTGGGACGCTCGAAGGCTTTAATTTGCAA<br>GCTTCGCAGTTTACACTCTCATCGTCGCTCTCATCATCGCTTCCGTTGTTGTTTTCC<br>TTAGTAGCGTCTGCTTCCAGAGAGTATTTATCTCTTATTACCTCTAAAGGTTCTGC<br>TTGATTTCTGACTTTGTTCGCCTCATGTGCATATTTTTCTTGGTTCTTTTGGGACAA<br>AATATGCGTAAAGGACTTTTGTTGTTCCCTCACATTCCA
ebs1	TATTGTTCGTTTTTTATCATAGTTTTTCAAATCTTCATATGAATACTAGGTAGAAA<br>TTTGGCTTATCAGTACGAATGAATTACAGTTTGACTGATTTGTACACGAGAATTG<br>ACGTATAACTTCTTTTGAAAGTCTTTGAAATTACATGGTAGTACATACATATATA<br>TATATATATATATATGTTATATTATTGTTAATTAAATATTATTATGCGTATTTTCCT<br>TTTCTTTATTAGTATAGTATTAATGACAGTAATAATAATAATAATAGTAACAATA<br>TCTCTTTTTTTTTTTCAGTGAGCTTTTATTTTTTTTTCATTGCTCTTCTTTTGGCCTC<br>TTTTGTTTTTTTCTTGATTTCCTCCAGTTTCATCTGTTTTTTCTTTGGATCAGATAC<br>AAAATCTGGTTTGAACGCGTCATA
efm1	ATTTAGCTTTGATCTGTAGCCTAAGTATAAAATTCTACGTATGTATATATTTACAT<br>GCAATTTTTTCTTTTTCCAATTCATGTTAATGTTCTTCATCATTTGATAATAGGCC<br>AATGATATTATCTACTATCTTCTCCTTTTGAGGAGAAGCTGCTTTCACTTCCTCGT<br>TGCTTTTCTCTTCGTATAAATACATGAATTTCGACTTGTCCAGCATTTTTTTATCA<br>ATACAATGCATGTGCCCATGAAGTTCTACCTCCGACAAATCTTTAACAAAATATG<br>TCGTTATTTGTGTCATGAATTTCAGTAGGGTTTGAAATATTTCGGGTATGGCGTTC<br>TTCTTATCCAGAATAACAATTTTGTAGTTTGCTGATAATGTTATTGGACGAAGCT<br>GGATGGGCCTATTT
ent2	TACTGTCCTATATATACTTTTTTGTCATGGGTACACATACATGTACGACATATATA<br>TATATATATATATATATATTAGCGAGTTTACATTGATGTAGAGCAGTAACTGGAA<br>GAAATAAAAATTTAAACTTAATACTAGGGGGTCCGTCTTCTCGTGGGTACTTCTT<br>TTACAGTTTTCAACTTCTGCTGACTTGTATGCCTTCTTGGCGCTTCTTCGGAAAAG<br>TTGACAGCGGGCTTTACTTTAACGGCAAATCGCAGTAAGTAAGCAAGTAACGAA<br>CTCGCACTTCAGCAAAATAAAAAAGAACAATCGATTAGAAATTCAAATTTCACC<br>GTGACTCTTTAACCCCTGCCGAATATTATAAGCAAACTATGATAACACTCTTATT<br>ATACCTGTGCGTAATATGTAACGCAATAGTGTTAATAAGGGCTGATTCGATAGCG
hbt1	ACACTTCTCGATTAACAAATTCCCAGTATTCTTTGAAATCTATTTTTCTTCCTCAA<br>TTGAATTTGAATAACTGTCTACGCGGACTCCTCCTATCTACAACTACAACAAATT<br>TTAACCACTTTATTACCACTTTCCTCTTTCATTTATTTTTGTCTTTTATGTTGTCAA<br>TTTACTAGTATTTTTTTTTTTTTCATTTACGTTCAAGGTTTTTATACTCATTTAAC<br>TTGTCTTAGGTTATTTATATATATACCTATATATTTATATATATATATATATATGT<br>ATGTATATATTATTATCACCAAATGAGAAATAATAGCTAATTTGATTTTTGATTAT<br>TTAAAATATTGGTTTGTTCTTTCTGCAAACATCTCGTTTGGTACGATATTAGTGAA<br>AAACGATGTAATTATCAACACGTGCATTACCCA
lsc2	TCTCGAGAAAAACAAAAGAGTTAATAATAAAGTATATATGCTTTTTTACTATTAA<br>TAAAGTTTCTTATTTTCCCTACATGTATACATACATATAAATATACTCTTTTGGAA<br>AAAATTTCTTTTTTTTTTTTTTAATTTTTTTATATTTTTCCATCTTTTGATGTAATAT<br>AGAGGTTATATAATGAAAGAATATATAAGAGGTCATAAAAAATCATTAAAAAAA<br>TGCGAGTTCAGGAACATATTATCGTTTACGTAATCAGTGCGTTATTGGCGGATTA<br>GTACCGATCACACCTTTGTTCATCGATGTGGATGCAGTTTGCTTTGCTGCGACGG<br>CTGCAGGAGTCTCCCCTTTTATTATGTAGTGAATAACCTGTTTAAAAGAATCACC<br>GTTCCGTATCCTAAAGTTAGCAATTCTTGTTAGATGGTAGCCTGCAGTGCAACCC<br>AGGAAAAAAATTGACGGAAGCTAACAGATAGTTCTTGGGCTTGATGACAAACGAC<br>CAA

prx1	AAATAAGCTTTGAAATAAACGACTTTACTATATACAGGTATATGAAGTATCTCTA<br>ACTAAAACTTTTATCTATCTTTCATTCTTATTATATCTCATCTCGTACGAAGGGCC<br>GCTCATTGGATCATTTTTCTTTACATACCGTAAAGGAATGGCGTTAAAAATATAT<br>ATATGAAAATGCATGTAATAAACTCTCTGCAGAAACCTTAATGTCAAAGGTCCCG<br>TATACAGATTATATTGGCTCTGCGTATACGCATTCTCGTCATGGTGAAGGATAAT<br>CGAGATTCTGACCAAGACCAAGATTTTAGTTCTGCTCACATGAAAAGACAACCG<br>GAGCAGCAACAGTTGCAACAGCACCAGTTCCCAAGTAAGAAACAACGAATATCT<br>CACCATGATGACAGTCATCAAATCAACCATAGACCAGTTACCTCATGTACACATT<br>GTAGACAGCACAAAATCAAATGCGATG
rpl15a	TAAGCTGGTTGATGGAAAATATAATTTTATTGGGCAAACTTTTGTTTATCTGATGT<br>GTTTTATACTATTATCTTTTTAATTAATGATTCTATATACAAACCTGTATATTTTTT<br>CTTTAACCAATTTTTTTTTTTATAGACCTAGAGCTGTACTTTTATTCTGCTATCAA<br>GCAAACCCCTACCCCCTCTTCTCAATCCTCCCCTCAGGCAGAACTTATCTACCTGT<br>ATCAAGGAGCGGACGAGGGAGTCCTAATTGTTCTACGTATACCAATGCTAGCAG<br>CTTACATAGGTGGTGGCACTACCATAATACTTATATATAATATCTTTACGCTCAA<br>GTAAATTTCTCCGCCGCATAAATTAACACCATGTTCTTTTTAGTCGCACCTTATTT<br>TCTTGTATTTTTTTTCTTTCCTTTTCTTCCCGTTTTTCC
rpl41b	GCGGATTGAGAGCAAATCGTTAAGTTCAGGTCAAGTAAAAATTGATTTCGAAAA<br>CTAATTTCTCTTATACAATCCTTTGATTGGACCGTCATCCTTTCGAATATAAGATT<br>TTGTTAAGAATATTTTAGACAGAGATCTACTTTATATTTAATATCTAGATATTACA<br>TAATTTCCTCTCTAATAAAATATCATTAATAAAATAAAAATGAAGCGATTTGATT<br>TTGTGTTGTCAACTTAGTTTGCCGCTATGCCTCTTGGGTAATGCTATTATTGAATC<br>GAAGGGCTTTATTATATTACCCTTTAGCTTATTCTGAGGTTTCTGTGGCGTGCAAA<br>GTGATGAACCGGGCGGGTTTTAAGGATAAAATCAAAAAGTGAAAAAATGAACG<br>GAAAATGGAATACCTGTGAAATGGAGAATGATAATGAATCTTTCTGTCGTGCTTG<br>AAAGATTTTCGGCT
rpp2b	ATAAACAATTTTCTTGTTTTCCTTCATTTTACAAAATCTGGTGTAAATCCTCGATT<br>CCATTGTGATTTAAAAATAATTAATTACTGTATAAGATTCTATATAGACAATTAA<br>TTGAAAATAGTATTACTTCTTTAACTCGCTTAGGATTTTTTTTATTTTTTCTCTCTT<br>TTCCGGAGAATTGCCAAATGTTTTCACGGCCTCTTCCAATAATAGCTTCATTTCTT<br>CCAACGCAACTACTAGTTTCCCATTGTAAGTGGCGAAGTACTCGCTGATATCATC<br>ATCTTCAGAAGCTTCTTTAATGGAAGTTGTTAAAGAATTTATAGAATTTGTTTGG<br>CTCAGAATATCTGATAATTTACTTTCGCCTATTTGGAGAGCCTGACATTTCAGTAT<br>TAAATCTTTCCTTAAATTACTATACTCGCTTAGAACTTTGAGATCATTTTCCACTT<br>CAACTTCCTCTTCGTCTGCTACTTCCATCTGCGGCAGCTTCAACACATCT
rps20	AACTAAGCTGGTTCTAACTGGAAATAATTTCCATTAGATTCCTCTTTTTCTCGTCC<br>ATTAACCAAAATATATTATTGAATTCAGCGGTTCCTTTTTTCTCATTTTCGCATAT<br>AGCTGCACTATTAGAATCAGCCCACTCTAGGTAAACACAGTTCCTCGATATACCT<br>CTGTCTTACTATCAGTGGTTAAACCTTATGCAAATATAATATATATATATATATAT<br>ATATATATATCTCATACTTTTGTTGATTCTTGTGTAATTATTGGAAAAGACAAAAC<br>AAAGCAAGCGTTTCTATTCATATTTACAAGTATTTTTTATGACAAACTATTTCTTA<br>ATTTTCCCACCGGCGGCTTTGAATAAGGCAATGTCATTGTCCTGCATAATATATT<br>GTTTGCCTGCACGTTTGATAAGTCCCTTAGATTTTAGTAAAGACTCATTTAGCGGT<br>GGTTCCATCTTC
trm82	TCGTGAAATAAAAAGGTAGTAAGTACATCTTTATAACTAATATAAAAAAAGATA<br>CGTACCGATTTGTGTTAATAATCATTGTTAGTACATCTTTATACCTCTGTTTCTAG<br>CTGAAGGCGGCGAATTGAATAGCAGTTCTTTTTAAATTGGCAGATACAATAGGAT<br>TAATCTCATTGATACATTTTTGTAGTCTATTTGTGTCGTTCTGGAAACAGTTTTGT<br>AAGCACGCCCTTAATGTGGCCTCGGTGTCCTTTTCAAGTAAAGGACCCATAACCT<br>TCTGAAAGAATTCCAAATCCACTATAATTTGTAATGAGCCATCATTAGAAAGATT<br>ACCAACATAAGGCTTAAATGCCTCAAAGAGGTATCTGGCTATAAAAATCTGCGTT<br>TCAATTAAAATCTTATGGATGAGCTGAGGCCCAATTCTAAAGCACTCACTGTGAA<br>CCACG

vps1	GTTTTCCTCATCTATACCGGTCGACTCAAGCTTGGTTTTGAGTATTTCTCCCTAAT<br>ATTTACTGCAAATCTTATATATTGTTCTTGTATACGTGGAAGCTAGTCTATAAATT<br>ATGCTTTTGAAGAATTTCCTATCTCGCATTACGATCCTAGTATCTTTTGTCCAATA<br>TGAAGAAGGTCAACATGAGGATGGGAATGATAATTGATAGCATATAATATTATT<br>CTTTTGTCAATACTAGTGTTTAGGATATTTCTACTAATACCTAATACCTCAATGGT<br>CCAATACTAAATAAGGTACTATTCATTGTATTGATTGATTCTGCATTTATCTTTCT<br>CTTGAACCGTAAATATCATTTTCATAAGTCACATGATAAAAACATATTTAAAATT<br>TTAAAAAAATTAATTTTCAAAATAAATTTATTATATTTTTTTAATTACATAATCAT<br>AAAAATAAATGTTCATGATTTCCGAACGT
yip5	AAATATGCGCACGTCTATACACACAATACTTTTTATGACTAGTATCATTGTATAC<br>TCTTATTATGTAGGGTTATACATGAGTGTTTATACGTTTTCTGTGTGTACATATAT<br>ATCCATATATGTTAATGCTAAATGCAAGTTTTGTGATGCTATTCATTAGAGATGC<br>CTTTGTCTAAAAATCAATGCTTTTATAGTCATCATAGGTTATGTCGTAAATGGTTT<br>TATCCTTAAATTTTAAGGAGATTTTGGATGAAATGTTTATGTCAGCTCTGGTAAA<br>GCCTTTTGAAAAAGTACATTTATCATTTTTTGGGAAATTAGTGGAAGAAGAAGAA<br>TGCTTTTTCTTAATGCATGGTAAACAAGGTGTGAGCAAAACCAACTTATCAATAT<br>GCTTAATCAACTTGCGTGAGCCTGTACCCTCAATGACATCATCCGGTATGGCAAT
ypr1	TGAGCGCGCTACATTACAGTTAATGCCTCCAGCAACCTGTAGTGCTTCTTTAAAC<br>CGATCCGATACAGCTGAAACAAACATTGATTTTACTAATTTTCATATAATATATA<br>CACATATATCGAATGATAATGCAAAATATAATTTAATGAAATTGGAAATACTCG<br>GAAAATTTATCATTATCCTTTTCTTCTTTTGAACGTAAACTTCGGACCGTCGTCAT<br>CGTCGTCGTCGCCATCACCGTTATTAGAAGCCCCAAAAGAGCCACGTGATCTACT<br>AGTTGCGCTTCTACTGTGTCTACTTTGCTTGGAACATTCTTCTGACAAGTTTGGCC<br>GTAAGTTCATTTCCTTCACCACAAAAAGATTACGATTATTTTCTCTGTGATTTAGT<br>CTATCGCTTTTATCATCAATATCACCCAGATCATCTTCATCAGTAATAAAAATTTT<br>GTTTTTTAGTGGTTGAACGCTGGCTGCAGTACCTGAACA
ypt31	GAGATATTTTGCAGCAGTTGCGCACTTGCATGTGAATGACTCTTCTCCCCTTTAAT<br>TCTGTGCTATATTTTTACAATTTTCTGCTGACATATAGTTTATATACATATAGAAC<br>GCATATAGGAAATTGAAGTAAACAGAATACACAAGTAGAGGCCGGTATGTACGA<br>CATTTTGCTTACTACTCTTTAAAATCATCGTCTTCTTCGTCTTCATCGTCTTCTTCT<br>TTTTCACCATATCCTACATCATCTTTAGAGCCTGTGCTAGGTTCCTTCTTGTCTAA<br>TTCTTCTGCAGTCTTTTTATAGTCAATTACTTTGCCGCGTGTTCTTCTTCCGGATGT<br>GATGATATTAGAGGTATCAATTTCTGCCAAATCGTCCTCTTCTTCTTCTCCCTCAT<br>TTCCCATCAATGCGTCTAACTTGGCATCGTCCATATCAGA

Supplementary Table 3. UniProt IDs of ORFs used in this study.

Source Organism	Gene name	UniProt ID
Aspergillus fumigatus	*dmaW*	Q50EL0
Aspergillus fumigatus	*easF*	Q4WZ60
Claviceps purpurea	*easC*	M1WA44
Aspergillus fumigatus	*easD*	Q4WZ66
Claviceps purpurea	*easG*	M1WEN5
Aspergillus japonicus	*easE*	-
Neotyphodium lolii		S5TDB9
Epichloe funkii		R9W1Q5
Periglandular ipomoeae		G9FM46
Epichloe elymi		K0HDR9
Epichloe inebrians		I7DFY5
Claviceps fusiformis		A8C7R9
Aspergillus lentulus		A0A0S7DKV5
Epichloe coenophialia		R9W261
Neotyphodium lolii	*easA*	S5TCI4
Periglandular ipomoeae		G9FM51
Epichloe ecoenophialia		R9W1H0
Claviceps purpurea		M1W0Y0
Botrytis cinerea	*cloA*	M7UEF9
Claviceps fusiformis		A8C7R4
Claviceps paspali		G8GV80
Claviceps purpurea		M1WEN7
Claviceps purpurea 20.1		M1VZB1
Claviceps purpurea 20.1		M1W6N5
Colletotrichum gloeosporioides		T0KET3
Colletotrichum gloeosporioides		T0KX90
Epichloe coenophiala		R9W253
Epichloe coenophiala		R9VXN6
Neotyphodium lolii		S5SWI2
Metarhizium acridum		E9EAT5
Metarhizium acridum		E9DSJ7
Metarhizium robertsii		E9F0X7
Metarhizium robertsii		E9F392
Periglandula ipomoeae		G9FM49
Saccharomyces cerevisiae	*fad1*	P38913
Saccharomyces cerevisiae	*pdi1*	P17967

Supplementary Table 4. Summary of the exact mass and retention time of chanoclavine-I detected from the screen of EasE orthologues in positive mode with an electrospray ionization source (ESI).

EasE source	Calculated $[M+H]^+$ (m/z)	Observed $[M+H]^+$ (m/z)	Mass error δ (ppm)	Retention time (s)	Molecular formula [M]
A. japonicus	257.1648	257.1644	-1.56	120.6	$C_{16}H_{20}N_2O$
E. coenophalia	257.1648	257.1681	12.83	120.6	

Supplementary Table 5. Summary of the exact mass and retention time of agroclavine detected from the screen of EasA orthologues in positive mode with an electrospray ionization source (ESI).

Construct	Calculated $[M+H]^+$ (m/z)	Observed $[M+H]^+$ (m/z)	Mass error δ (ppm)	Retention time (s)	Molecular formula [M]
Agroclavine standard	239.1543	239.1496	-19.65	150.8	$C_{16}H_{18}N_2$
EasA_Pi	239.1543	239.1486	-23.83	151.3	
EasA_Nl	239.1543	239.1505	-15.89	151.4	
EasA_Cpur	239.1543	239.1502	-17.14	151.5	
EasA_Ec	239.1543	239.1507	-15.05	151.3	

Supplementary Table 6. Summary of the exact mass and retention time of DLA detected from the screen of CloA orthologues in positive mode with an electrospray ionization source (ESI).

**Construct**	**Calculated $[M+H]^+$ (m/z)**	**Observed $[M+H]^+$ (m/z)**	**Mass error, δ (ppm)**	**Retention time (s)**	**Molecular formula [M]**
500 nM DLA	269.1285	269.1260	-9.29	126.3	$C_{16}H_{16}N_2O_2$
CloA_Cpur	269.1285	269.1284	-0.37	128.1	
CloA_Cpas	269.1285	269.1261	-8.92	128.0	
CloA_Nlol	269.1285	269.1262	-8.55	128.2	
CloA_Pipo	269.1285	269.1287	0.74	128.1	
CloA_XN6	269.1285	269.1268	-6.32	128.2	

Supplementary Table 7. Summary of the exact masses measured for the detectable intermediates of the ergot alkaloid pathway with and without the incorporation of ^{13}C-2-indole-L-tryptophan, in positive mode with an electrospray ionization source (ESI).

Detected compounds	Calculated $[M+H]^+$ (m/z)	Observed $[M+H]^+$ (m/z)	Mass error δ (ppm)	Molecular formula [M]
DMAT	273.1598	273.1632	12.45	$C_{16} H_{20} N_2 O_2$
^{13}C-DMAT	274.1631	274.1623	-2.92	$C_{15}\, ^{13}C\, H_{20} N_2 O_2$
4DMA	287.1754	287.1749	-1.74	$C_{17} H_{22} N_2 O_2$
^{13}C-4DMA	288.1788	288.1784	-1.39	$C_{16}\, ^{13}C\, H_{22} N_2 O_2$
Chanoclavine-I	257.1648	257.1636	-4.67	$C_{16} H_{20} N_2 O$
^{13}C-Chanoclavine-I	258.1682	258.1668	-5.42	$C_{15}\, ^{13}C\, H_{20} N_2 O$
Agroclavine	239.1543	239.1527	-6.69	$C_{16} H_{18} N_2$
^{13}C-Agroclavine	240.1576	240.1572	-1.67	$C_{16}\, ^{13}C\, H_{18} N_2$

Supplementary Table 8. Summary values of calculated DLA titre from the strain DLAM33B in shake flasks. Statistical values were derived from three biological replicates.

**DLAM33B**	**[DLA] (nM)**	**[DLA] (μg/L)**
**Mean**	**266.42**	**71.48**
**95% Confidence Interval**		
**Upper limit**	**377.53**	**101.30**
**Lower limit**	**155.31**	**41.67**

Supplementary Table 9. List of yeast strains created in this study. All strains were derived from *S. cerevisiae* BY4741 as the base strain.

**Strain**	**Genotype**
YMWF	**Chr. XIII**:<br>*YMRWΔ15*:: P$_{TEF2}$-*dmaW*-T$_{ENT2}$/P$_{GPM1}$-*easF*-T$_{PRX1}$/P$_{GAL1}$-*yEGFP*-T$_{CBR1}$
YMC17	**Chr. XIII**:<br>*YMRWΔ15*:: P$_{TEF2}$-*dmaW*-T$_{ENT2}$/P$_{GPM1}$-*easF*-T$_{PRX1}$/P$_{GAL10}$-*easC*-T$_{YIP5}$/<br>P$_{CUP1}$-*mKOk*-T$_{CBR1}$
YOCE	**Chr. XIII**:<br>*YMRWΔ15*:: P$_{TEF2}$-*dmaW*-T$_{ENT2}$/P$_{GPM1}$-*easF*-T$_{PRX1}$/P$_{GAL1}$-yEGFP-T$_{CBR1}$<br>**Chr. XV**:<br>*YORWΔ22*::P$_{GAL10}$-*easC*-T$_{YIP5}$/P$_{GAL1}$-*easE_Aj*-T$_{RPS20}$
AgcM1B	**Chr. XIII**:<br>*YMRWΔ15*:: P$_{TDH2}$-*dmaW*-T$_{ENT2}$/ P$_{GPM1}$-*easF*-T$_{PRX1}$/ P$_{TEF2}$-*easE_Aj*-T$_{RPS20}$/<br>P$_{PGK1}$-*easC*-T$_{YIP5}$/ P$_{CUP1}$-*mKOk*-T$_{CBR1}$
AgcM2B	**Chr. XIII**:<br>*YMRWΔ15*:: P$_{TDH2}$-*dmaW*-T$_{ENT2}$/ P$_{GPM1}$-*easF*-T$_{PRX1}$/ P$_{TEF2}$-*easE_Aj*-T$_{RPS20}$/<br>P$_{PGK1}$-*easC*-T$_{YIP5}$/ P$_{CUP1}$-*mKOk*-T$_{CBR1}$<br>**Chr. III**:<br>*ARS308*:: P$_{RPL3}$-*PDI1*-T$_{VPS1}$/ P$_{PDA1}$-*easD*-T$_{YPR1}$/ P$_{ACT1}$-*FAD1*-T$_{EFM2}$/<br>P$_{RPL15}$-*easC*-T$_{YIP5}$/ P$_{TPI1}$-*dmaW*-T$_{ENT2}$
AgcM33B	**Chr. XIII**:<br>*YMRWΔ15*:: P$_{TDH2}$-*dmaW*-T$_{ENT2}$/ P$_{GPM1}$-*easF*-T$_{PRX1}$/ P$_{TEF2}$-*easE_Aj*-T$_{RPS20}$/<br>P$_{PGK1}$-*easC*-T$_{YIP5}$/ P$_{CUP1}$-*mKOk*-T$_{CBR1}$<br>**Chr. III**:<br>*ARS308*:: P$_{RPL3}$-*PDI1*-T$_{VPS1}$/ P$_{PDA1}$-*easD*-T$_{YPR1}$/ P$_{ACT1}$-*FAD1*-T$_{EFM2}$/<br>P$_{RPL15}$-*easC*-T$_{YIP5}$/ P$_{TPI1}$-*dmaW*-T$_{ENT2}$<br>**Chr. XVI**:<br>*YPRCΔ15*:: P$_{RPL8B}$-*easG*-T$_{APL2}$/ P$_{SSA1}$-*easA_Ec*-T$_{YPT31}$/ P$_{TEF2}$-*easF*-T$_{PRX1}$/<br>P$_{TDH2}$-*easC*-T$_{YIP5}$/P$_{TDH3}$-*easD*-T$_{YPR1}$
DLAM33B	**Chr. XIII**:<br>*YMRWΔ15*:: P$_{TDH2}$-*dmaW*-T$_{ENT2}$/ P$_{GPM1}$-*easF*-T$_{PRX1}$/ P$_{TEF2}$-*easE_Aj*-T$_{RPS20}$/<br>P$_{PGK1}$-*easC*-T$_{YIP5}$/ P$_{CUP1}$-*mKOk*-T$_{CBR1}$<br>**Chr. III**:<br>*ARS308*:: P$_{RPL3}$-*PDI1*-T$_{VPS1}$/ P$_{PDA1}$-*easD*-T$_{YPR1}$/ P$_{ACT1}$-*FAD1*-T$_{EFM2}$/<br>P$_{RPL15}$-*easC*-T$_{YIP5}$/ P$_{TPI1}$-*dmaW*-T$_{ENT2}$<br>**Chr. XVI**:<br>*YPRCΔ15*:: P$_{RPL8B}$-*easG*-T$_{APL2}$/ P$_{SSA1}$-*easA_Ec*-T$_{YPT31}$/ P$_{TEF2}$-*easF*-T$_{PRX1}$/<br>P$_{TDH2}$-*easC*-T$_{YIP5}$/ P$_{TDH3}$-*easD*-T$_{YPR1}$<br>**Chr. II**:<br>*ARS208*:: P$_{GAL1}$-cloA_Cpur-T$_{RPL41B}$

Supplementary Table 10. Composition of 10X PBS solution, used for the preparation of 1X PBS (pH 7.4) for screening of *cloA* orthologs.

Component	Mass (for 1 L solution)	Molarity
NaCl (mw: 58.4 g/mol)	80 g	1.37 M
KCl (mw: 74.551 g/mol)	2 g	0.027 M
Na_2HPO_4 (mw: 141.96 g/mol)	14.4 g	0.1 M
KH_2PO_4 (mw: 136.086 g/mol)	2.45 g	0.018 M

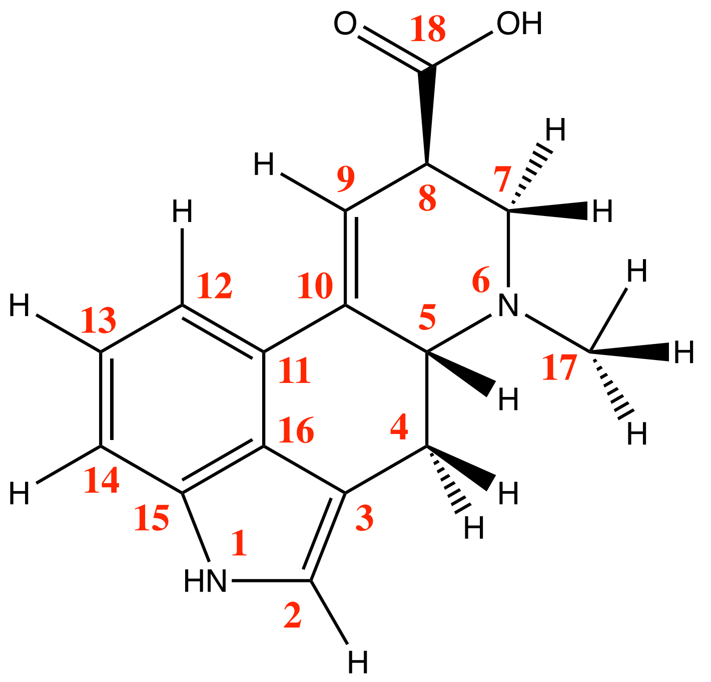

Supplementary Table 11. ^{1}H-NMR assignments for *D*-lysergic acid in D_2O.

**Position**	**^{1}H-NMR (Standard)**	**^{1}H-NMR (Sample)**
2	7.194 – 7.197	7.210 – 7.358$^{\alpha}$
4	2.964 – 3.008	2.913 – 2.984
5	4.006 – 4.040	3.455 – 4.099$^{\beta}$
7	3.349 – 3.395	3.455 – 4.099$^{\beta}$
8	4.030 – 4.040	3.455 – 4.099$^{\beta}$
12	7.225 –7.227	7.210 – 7.358$^{\alpha}$
13	7.110 – 7.142	7.112 – 7.129
14	7.209 – 7.213	7.210 – 7.358$^{\alpha}$
17	2.143	2.108

$^{\alpha\text{-}\beta}$: Broad indistinguishable peaks, chemical shifts with the same symbol overlap.

Supplementary Table 12. Summary of end-point DLA titres from 4 and 1 L fermentation of DLAM33B.

**DLAM33B**	**[DLA] (μM)**		**[DLA] (μg/L)**	
**Fermenter Scale**	**4 L**	**1 L**	**4 L**	**1 L**
**Mean**	**5.32**	**6.32**	**1427.85**	**1695.91**
**95 % Confidence Interval**				
**Lower limit**	**5.12**	**6.07**	**1373.96**	**1628.45**
**Upper limit**	**5.48**	**6.54**	**1470.21**	**1754.46**

Supplementary Table 13. Primer sequences used in this study. Primer sequences used to clone the promoters and terminators were designed by appending the following prefix sequence 5’-GGTCTCGGGCT-3’ (promoters) / 5’-GGTCTCGTAGC-3’ (terminators) and the suffix sequence 5’-GGTCTCGCATC-3’ (promoters) / 5’- GGTCTCGCCTC-3’ (terminators) to 25 bps of the start and end of these sequences to be cloned.

**p [M/C] K [U/L/H]-RFP Assembly**	
Name	5'- Sequence - 3'
path-RFP F'	CCCTGACTAATGCCGACGTCTCGACCTCGAGACCGCAATACGCAAACCGCCTCTC
HIS3 F'	GAGAGGCGGTTTGCGTATTGCGGTCTCGAGGTCGAGACGTCGGCATTAGTCAGGG
HIS3 R'	CACGTATAGAATGATGCATTACCTTGTCATCTTCAGTATCATACTGTTCGTATACA
Kan R'	TGTATACGAACAGTATGATACTGAAGATGACAAGGTAATGCATCATTCTATACGTG
Kan F'	GCACGTGATGAAAAGGACCCAGGTGGCATTGACTTGATCGGCACGTAAGAGGTTC
CEN R'	GAACCTCTTACGTGCCGATCAAGTCAATGCCACCTGGGTCCTTTTCATCACGTGC
Cen F'	CTACTAGAGTGGTCTCATGAGCGAGACGTCCGGCATCCGCTTACAGACAAGCTGT
path-RFP R'	ACAGCTTGTCTGTAAGCGGATGCCGGACGTCTCGCTCATGAGACCACTCTAGTAG
**pGAU-[int site]-RFP Assembly**	
Name	5'- Sequence - 3'
pGAU-ORI F'	GCAAAAGGCCAGGAACCGT
pGAU-ORI R'	GTGGCACTTTTCGGGGAAATG
YPRCd15up F'	ATTTCCCCGAAAAGTGCCACGGTCTCTGTCAGATAACGCC
YPRCd15up R'	TGCTTCGAGCCAGGTAGAGATTTGCGAAACCCTATGCTCTGT
YMRWd15up F'	ATTTCCCCGAAAAGTGCCACGGTCTCTGTCATCAATCAAAGCAACC
YMRWd15up F'	TGCTTCGAGCCAGGTAGAGAGCGGTGTAAGAAAATGACATAAAGTTTGA
YORWd17up F'	ATTTCCCCGAAAAGTGCCACGGTCTCTGTCAGTGCACAAAGGCCATAATATTATGT
YORWd17up F'	TGCTTCGAGCCAGGTAGAGAATGGCATGAGTTATGGTTGCACAGT
ARS208up F'	ATTTCCCCGAAAAGTGCCACGGTCTCTGTCAAAGGTACTAGGGCTGTTACC
ARS208up R'	TGCTTCGAGCCAGGTAGAGAGTGTGTATGGTCCCTATTGGCACCG
ARS308up F'	ATTTCCCCGAAAAGTGCCACGGTCTCTGTCATATTTCAGAAAAATTATTC
ARS308up R'	TGCTTCGAGCCAGGTAGAGATTAGATAAAAAGAAAAAAATTCGAA
pGAU-URR2 F'	TCTCTACCTGGCTCGAAGCA
HcK_O-URA3 F'	CTGGTCTCGGATGCATACCACAGCTTTTCAATTCAATTCATCA
HcK_O-URA3 R'	CTGGTCTCGGCTACCTGATGCGGTATTTTCTCCTTACG
pGAU-URR2 R'	TCTCTGCCTGAGGTGGTGC
pGAU-RFP F'	TGCACCACCTCAGGCAGAGAACCTAGAGACGGCAATACGCAAACCGCCTCT
pGAU-RFP R'	CTCATGAGACGGAGCCAGTGTGACTCTAGTAGAGAG
YPRCd15down F'	CACTGGCTCCGTCTCATGAGCGCTTGGAAGGTCGGGATG
YPRCd15down R'	TACGGTTCCTGGCCTTTTGCGGTCTCTCCGACATAAAGCAG
YMRWd15down F'	CACTGGCTCCGTCTCATGAGCGCTCATGGAAAATGCAACCG
YMRWd15down F'	TACGGTTCCTGGCCTTTTGCATCGGTCTCTCCGAGCCG

YORWd17down F'	CACTGGCTCCGTCTCATGAGCGTCCCCAACAAAAGTGGGCTCTCA
YORWd17down F'	TACGGTTCCTGGCCTTTTGCATCGGTCTCTCCGATCTTAAAGCTGGCTCCC
ARS208down F'	CACTGGCTCCGTCTCATGAGACTTGTAGTTTATGTGCTTTATAGT
ARS208down R'	TACGGTTCCTGGCCTTTTGCATCGGTCTCTCCGAATTAATTAATAAATTTAAATAG
ARS308down F'	CACTGGCTCCGTCTCATGAGTCTTTGCTACATATTGCTACCACTT
ARS308down R'	TACGGTTCCTGGCCTTTTGCATCGGTCTCTCCGATGATAGAACGAGTACAAC
**ORF primers**	
Name	5'- Sequence - 3'
dmaW_forGG	ATTAGGTCTCGGATGCACCATCACCATCACCACAT
EasF_forGG	ATTAGGTCTCGGATGACCATCTCTGCACCGCCGAT
dmaW_RevGGTag	ATTAGGTCTCGCAGCTCAATGTAAACCAGAG
EasF_RevGGTag	ATTAGGTCTCGCAGCGTTCAGACGCAGGCGCAG
EasD_forGG	ATTAGGTCTCGGATGGCATCAGTGGAATCGCGTAT
EasD_RevGG	ATTAGGTCTCGGCTACGGCATACAGGCACCCA
EasG_forGG	ATTAGGTCTCGGATGACAGTATTATTAACAGGTGG
EasG_RevGG	ATTAGGTCTCGGCTACTTTCTTGCTCTCCATGCTGCC
easAPi_GGfwd	ATTAGGTCTCGGATGTCTACTTCCAACTTGTTCAACGAATTG
easAPi_GGrev	ATTAGGTCTCGCAGCAGCAACAGTAGTTTCTTGGTCACC
easANl_GGfwd	ATTAGGTCTCGGATGTCTACTTCTAACTTGTTCACCCCATTGCAA
easANl_GGrev	ATTAGGTCTCGCAGCAGCCAAAACAGCTTGCTTCTTGTTTTCAGT
easAEI_GGfwd	ATTAGGTCTCGGATGTCTACCTCTAACTTGTTCAACGCTT
easAEI_GGrev	ATTAGGTCTCGCAGCGTTCTTTCTCATAGAAGCAGTAGTAT
easACP_GGfwd	ATTAGGTCTCGGATGTCAACTTCTAATTTGTTTAATAC
easACP_Ggrev	ATTAGGTCTCGGCTAACCTGCAACAGCAGCTTCATCAGC
PDI1_forGG	ATTAGGTCTCGGATGAAGTTTTCTGCTGGTGCCGTCC
FAD1_forGG	ATTAGGTCTCGGATGCAGTTGAGCAAGGCTGCTGAGATGTG
PDI1_RevGG	ATTAGGTCTCGGCTACAATTCATCGTGAATGGCATCTTCTTCG
FAD1_RevGG	ATTAGGTCTCGGCTAATTCTTGATCCTGCCTGCTCTCTCTAAAG
FAD1_bsmBI F'	CGCATTTAGAGATTTTATAAAGATATACCCTGAAACTGAAGCTATAGTGATAGGTATTAGACACACAG
FAD1_bsmBI R'	CTGTGTGTCTAATACCTATCACTATAGCTTCAGTTTCAGGGTATATCTTTATAAAATCTCTAAATGCG

SUPPLEMENTARY REFERENCES

1 Gerlt, J. A. *et al.* Enzyme function initiative-enzyme similarity tool (EFI-EST): A web tool for generating protein sequence similarity networks. *Biochimica Et Biophysica Acta (BBA)-Proteins and Proteomics* **1854**, 1019-1037 (2015).

2 Zallot, R., Oberg, N. O. & Gerlt, J. A. 'Democratized'genomic enzymology web tools for functional assignment. *Current opinion in chemical biology* **47**, 77-85 (2018).

3 Frickey, T. & Lupas, A. CLANS: a Java application for visualizing protein families based on pairwise similarity. *Bioinformatics* **20**, 3702-3704 (2004).

4 Cheng, S. *et al.* Sequence similarity network reveals the imprints of major diversification events in the evolution of microbial life. *Frontiers in Ecology and Evolution* **2**, 72 (2014).

5 Shannon, P. *et al.* Cytoscape: a software environment for integrated models of biomolecular interaction networks. *Genome research* **13**, 2498-2504 (2003).

6 Zallot, R., Oberg, N. & Gerlt, J. A. The EFI Web Resource for Genomic Enzymology Tools: Leveraging Protein, Genome, and Metagenome Databases to Discover Novel Enzymes and Metabolic Pathways. *Biochemistry* **58**, 4169-4182, doi:10.1021/acs.biochem.9b00735 (2019).

7 Nielsen, C. A. *et al.* The important ergot alkaloid intermediate chanoclavine-I produced in the yeast Saccharomyces cerevisiae by the combined action of EasC and EasE from Aspergillus japonicus. *Microbial cell factories* **13**, 1 (2014).

8 Cheng, J. Z., Coyle, C. M., Panaccione, D. G. & O'Connor, S. E. Controlling a structural branch point in ergot alkaloid biosynthesis. *Journal of the American Chemical Society* **132**, 12835-12837 (2010).

9 Guo, Y. *et al.* YeastFab: the design and construction of standard biological parts for metabolic engineering in Saccharomyces cerevisiae. *Nucleic acids research* **43**, e88-e88 (2015).

10 Zhu, J. & Zhang, M. Q. SCPD: a promoter database of the yeast Saccharomyces cerevisiae. *Bioinformatics* **15**, 607-611 (1999).

11 Redden, H. & Alper, H. S. The development and characterization of synthetic minimal yeast promoters. *Nature communications* **6**, 7810 (2015).

12 Partow, S., Siewers, V., Bjørn, S., Nielsen, J. & Maury, J. Characterization of different promoters for designing a new expression vector in Saccharomyces cerevisiae. *Yeast* **27**, 955-964 (2010).

13 Lee, M. E., DeLoache, W. C., Cervantes, B. & Dueber, J. E. A highly characterized yeast toolkit for modular, multipart assembly. *ACS synthetic biology* **4**, 975-986 (2015).

14 Yamanishi, M. *et al.* A genome-wide activity assessment of terminator regions in Saccharomyces cerevisiae provides a ″terminatome ″toolbox. *ACS synthetic biology* **2**, 337-347 (2013).

15 Gibson, D. G. *et al.* Enzymatic assembly of DNA molecules up to several hundred kilobases. *Nature methods* **6**, 343-345 (2009).

16 Pundir, S., Martin, M. J., O'Donovan, C. & Consortium, U. UniProt tools. *Current protocols in bioinformatics* **53**, 1.29. 21-21.29. 15 (2016).
